# Gut‐Derived Exosomes Mediate the Microbiota Dysbiosis‐Induced Spermatogenesis Impairment by Targeting Meioc in Mice

**DOI:** 10.1002/advs.202310110

**Published:** 2024-03-25

**Authors:** Tong Chen, Boqi Zhang, Guitian He, Nan Wang, Maosheng Cao, Caomeihui Shen, Xue Chen, Lu Chen, Kening Liu, Yuxin Luo, Yiqiu huang, Chenfeng Yuan, Xu Zhou, Chunjin Li

**Affiliations:** ^1^ College of Animal Sciences Jilin University 5333 Xian Road Changchun Jilin 130062 China

**Keywords:** exosomes, gut microbiota dysbiosis, meiosis, miR‐211‐5p, spermatogenesis

## Abstract

Diseases like obesity and intestinal inflammation diseases are accompanied by dysbiosis of the gut microbiota (DSGM), which leads to various complications, including systemic metabolic disorders. DSGM reportedly impairs the fertility of male mice; however, the regulatory mechanism is unclear. Exosomes are molecular mediators of intercellular communication, but the regulation of spermatogenesis by non‐reproductive tissue‐originated exosomes remains unknown. The present study shows that DSGM altered the miRNA expression profile of mouse circulating exosomes and impaired spermatogenesis. Moreover, the single‐cell sequencing results indicate that circulating exosomes from mice with DSGM impaired spermatogenesis, while circulating exosomes from wild mice improved spermatogenesis by promoting meiosis. Further study demonstrates that DSGM leads to abnormal upregulation of miR‐211‐5p in gut‐derived circulating exosomes, which inhibited the expression of meiosis‐specific with coiled‐coil domain (Meioc) in the testes and impaired spermatogenesis by disturbing meiosis process. In summary, this study defines the important role of gut‐derived exosomes in connecting the “gut‐testis” axis.

## Graphical abstract

This study proposes a new concept of the gut exosome testis axis. Following dysbiosis of gut microbiota, miR‐211‐5p loaded in the gut‐derived circulating exosomes is increased abnormally and transported to the testicular seminiferous tubules through the blood circulation, targeting and inhibiting the expression of Meioc, consequently disturbing the meiosis process and impairing spermatogenesis.

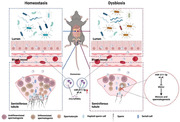

## 1 Introdution

Approximately 10–15% of couples suffer from infertility globally, and ≈50% of these fertility problems are caused by male infertility.^[^ ^1^ ^]^ Recent research showed that the average sperm count in men worldwide has decreased by 62% in the past 50 years.^[^ ^2^ ^]^ Several factors lead to male infertility, including congenital, acquired, or idiopathic factors, which damage spermatogenesis.^[^ ^3^ ^]^ Increased environmental pollutants can interact with the endocrine system and are closely associated with DSGM and the development of reproductive disorders, metabolic diseases, and other diseases.^[^ ^4^ ^]^

Recent studies showed that the composition of the human microbiota is closely related to human health.^[^ ^5^ ^]^ High‐fat diets (HFD) induce DSGM and endotoxemia in mice. Analysis of the intestinal microbiome from clinical subjects showed that the abundance of *Prevotella* bacteria was strongly negatively correlated with sperm vitality, and blood endotoxin levels were positively correlated with the abundance of *Bacteroides*.^[^ ^6^ ^]^ Bacterial translocation may lead to excessive activation of immune responses and a chronic inflammatory state, leading to endothelial damage, destruction of the blood‐testis barrier, and impairment of spermatogenesis and sperm vitality.^[^ ^7^ ^]^ The activation of the immune system by DSGM and bacterial translocation caused inflammation of the testes and epididymides and induced insulin resistance and resistance to gastrointestinal hormones such as leptin and ghrelin. This affected the secretion of various reproductive hormones such as follicle‐stimulating hormone, luteinizing hormone, and testosterone and regulated spermatogenesis.^[^ ^8^ ^]^ However, the molecular mediators and mechanisms through which gut microbiota affect male fertility are unknown.

Exosomes are extracellular vesicles between 30 and 160 nm in size and are released from cells through a specific, multi‐step exocytosis process.^[^ ^9^ ^]^ Exosomes from different sources usually contain a large number of specific lipids, proteins, and nucleic acids.^[^ ^10^ ^]^ Exosomes are transported by blood circulation to distal cell sites and enter cells through endocytosis, enabling their intracellular functions.^[^ ^11^ ^]^

Recently, exosomes have been identified as carrier of intercellular communication, and reportedly play important roles in stimulating the T cell response,^[^ ^12^ ^]^ participating in macrophage antigen presentation, regulating insulin sensitivity,^[^ ^13^ ^]^ regulating gametogenesis, embryogenesis, embryonic development, and other biological processes.^[^ ^14^ ^]^ Previous studies have shown that the Sertoli cell‐derived exosomes can enter the seminiferous tubules to regulate spermatogenesis. The exosomes in seminal plasma play an important role in sperm maturation, capacitation, acrosome reaction, and fertilization.^[^ ^15^ ^]^ However, the role of exosomes originating from non‐reproductive tissues in regulating male reproduction remains unclear.

The present study showed that DSGM significantly altered the expression profile of microRNAs (miRNAs) in the intestine and intestine‐derived circulating exosomes, resulting in the up‐regulation of miR‐211‐5p and down‐regulation of miR‐122‐5p. Additionally, abnormally increased miR‐211‐5p in gut‐derived exosomes apparently mediated microbiota dysbiosis‐induced spermatogenesis impairment by targeting Meioc in mice. These data identified the “gut‐exosomes‐testes axis” in which exosomes from non‐reproductive tissue (intestine) act as key molecular mediators for gut microbiota to regulate spermatogenesis in male animals.

## 2 Results

### 2.1 Antibiotic‐Induced Dysbiosis of Gut Microbiota Impairs Fertility in Mice

**Figure 1:**
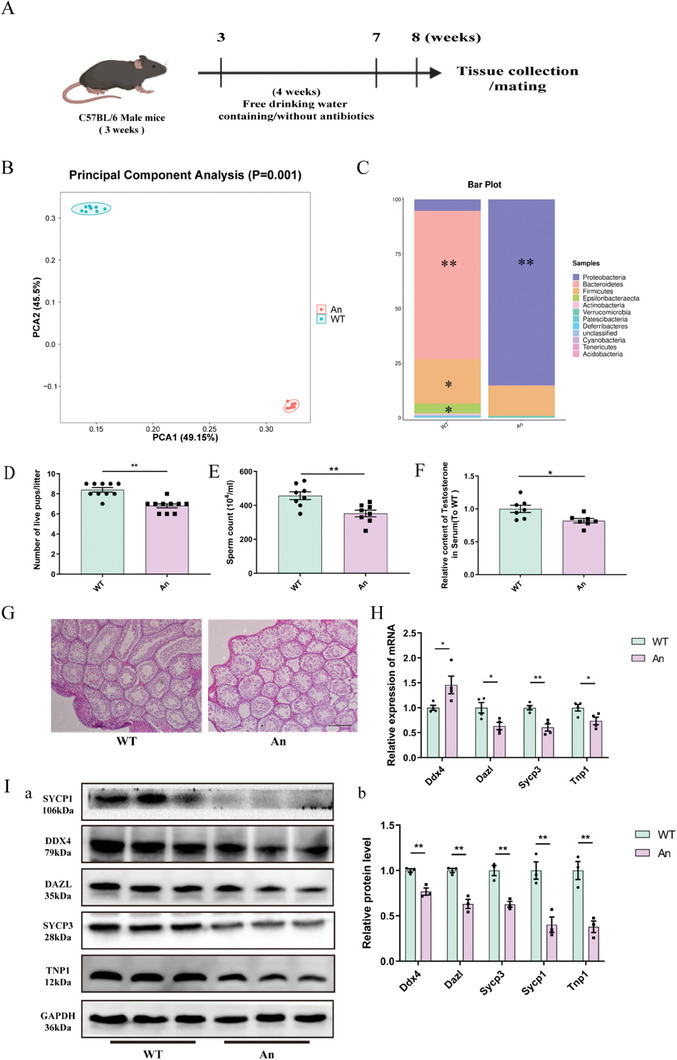
Effect of antibiotic‐induced dysbiosis of the gut microbiota on spermatogenesis in mice. (Data are expressed as means ± SEM.). A) Experimental scheme. B) Principal component analysis (PCA) plot of the gut microbiota based on the operational taxonomic unit metrics of the samples in the WT and An groups (*n* = 8). C) Bacterial taxonomic profiling at the phylum level of gut bacteria from different treatment groups (*n* = 8). D) Statistical analysis of litter size (*n* = 8). E) Statistical analysis of sperm count (*n* = 8). F) ELISA analysis of serum testosterone levels in the different treatment groups (*n* = 7). G) Representative testicular sections from the WT and An groups stained with H&E. Scale bar = 200 µm. H) mRNA quantification by qPCR in testes (*n* = 4). I) Western blotting a) and quantitative b) analysis of proteins.

To determine the impact of gut microbiota dysbiosis on male mice reproduction, an antibiotic‐induced DSGM model was established (**Figure** **1**A). The 16S‐rDNA sequencing analysis showed that antibiotics induced an imbalance of intestinal microbiota, resulting in differences in the abundance and composition of microbiota between the antibiotic treatment group (An) and the wild type (WT) control group (Figure 1B; Figure S1A, Supporting Information). Additionally, at the phylum level, antibiotic treatment led to a significant increase in the abundance of *Proteobacteria* and a significant decrease in the abundance of *Epsilonbacteraeota*, *Firmicutes*, and *Bacteroidetes* (Figure 1C; Figure S1B, Supporting Information). The weight of mice in the An group was significantly lower than that of mice in the WT group in the first three weeks, but the weights improved in the fourth week (Figure S1C, Supporting Information). This may be related to a decrease in water intake in the first week. These results indicated that the mouse model of DSGM was successfully established.

Notably, the results of fertility evaluation showed that compared with the WT group, the number of sperm in the epididymides, the litter sizes, and the level of serum testosterone in the An group were significantly decreased (Figure 1D–F). However, there was no significant difference in testicular weights between the two groups, nor in the pregnancy rate of the female mice (Figure S1D,E, Supporting Information). The expression level of DEAD‐box helicase 4 (Ddx4), deleted in azoospermia‐like (Dazl), synaptonemal complex protein 1/3 (Sycp1/3) and transition protein 1 (Tnp1) was used to characterize the changes of total germ cells, spermatogonia, spermatocytes, and haploid sperm cells, respectively. The results showed that the number of spermatogonia, spermatocytes, and haploid sperm cells in the An group was significantly reduced compared with the WT group (Figure 1H,I). H&E staining of testicular tissue sections further confirmed the impairment of spermatogenesis caused by antibiotic treatment, which was exhibited as a loose structure of convoluted seminiferous tubules and a loss of germ cells in convoluted seminiferous tubules (Figure 1G). These results demonstrated that DSGM induced by antibiotics is harmful to spermatogenesis and the fertility of mice.

### 2.2 Homeostasis of Microbiota Maintains Spermatogenesis in Mice While Dysbiosis of Microbiota Impairs Spermatogenesis

**Figure 2:**
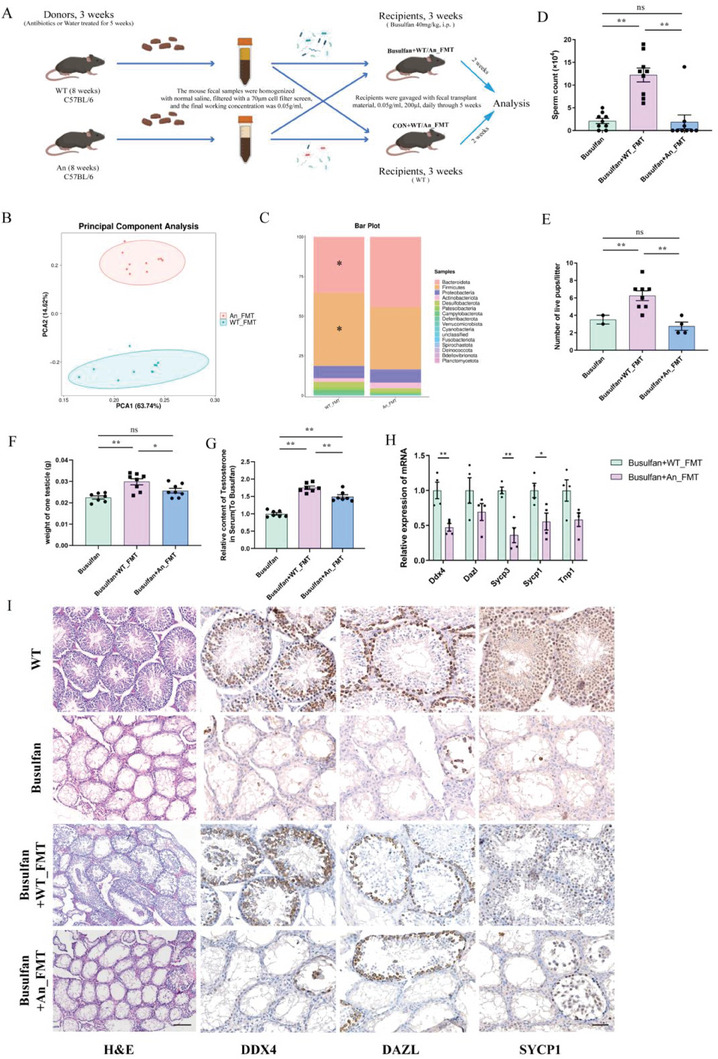
Effects of fecal microbiota transplantation (FMT) on spermatogenesis in busulfan‐treated mice. (Data are expressed as means ± SEM.). A) Experimental scheme. B) Principal component analysis (PCA) plot of the gut microbiota based on the operational taxonomic unit metrics of the samples in the CON+WT/An_FMT groups (*n* = 10). C) Bacterial taxonomic profiling at the phylum level of gut bacteria from CON+WT/An_FMT groups (*n* = 10). D) Statistical analysis of the sperm counts in busulfan + FMT‐treated mice (*n* = 9). E) Statistical analysis of litter sizes in busulfan + FMT‐treated mice (*n* = 8). F) Statistical analysis of the unilateral testes weights in busulfan + FMT‐treated mice (*n* = 8). G) ELISA analysis of serum testosterone in busulfan + FMT‐treated mice (*n* = 7). H) mRNA quantification using qPCR in busulfan + FMT‐treated mouse testes (*n* = 4). I) Representative testicular sections from busulfan + FMT‐treated mice with H&E and Immunohistochemistry (IHC) staining. H&E scale bar = 200 µm; IHC scale bar = 100 µm.

To exclude a direct impact of antibiotics on spermatogenesis and further identify the role of gut microbiota, fecal microbiota from the WT and An mice were transplanted to recipient mice (Fecal microbiota transplantation (FMT) was performed simultaneously both in wild‐type recipient mice and busulfan‐induced reproductive damage recipient mice to directly reflect the changes in mouse spermatogenesis process, the other models also serve the same purpose.) (**Figure** **2**A). The 16s‐rDNA sequencing results showed that there was no significant difference in the alpha diversity (species richness) of intestinal microbiota in the mice that received FMT from antibiotic‐treated mice (An_FMT) compared with the mice that received FMT from wild‐type mice (WT_FMT) (Figure S2A, Supporting Information). However, the An_FMT groups showed the same change as that of antibiotic treatment, showing a decrease in *Firmicutes* at the phylum level (Figures 1B,C and 2B,C; Figure S2B, Supporting Information). Compared with the WT_FMT group, the number of sperm in the epididymides, the litter sizes, and the pregnancy rate of the female mice, the weight of unilateral testes, and the levels of testosterone in the serum of mice decreased significantly in the An_FMT group (Figure 2D–G; Figure S2C–F, Supporting Information). The qPCR results of the testis tissues showed that the intracellular expression levels of marker genes were significantly reduced in the An_FMT group (Figure 2H; Figure S2G, Supporting Information). H&E and immunohistochemistry (IHC) staining of testicular tissue sections showed that numerous cavities developed in the convoluted seminiferous tubules of the An_FMT group and that the proportion of DDX4 positive convoluted seminiferous tubules and the number of spermatocytes decreased (Figure 2I; Figure S2H, Supporting Information). Therefore, DSGM impaired spermatogenesis and the fertility of mice, and FMT from WT mice alleviated the disordered spermatogenesis.

### 2.3 Circulating Exosomes Mediate DSGM‐Induced Reproductive Impairment in Mice

**Figure 3:**
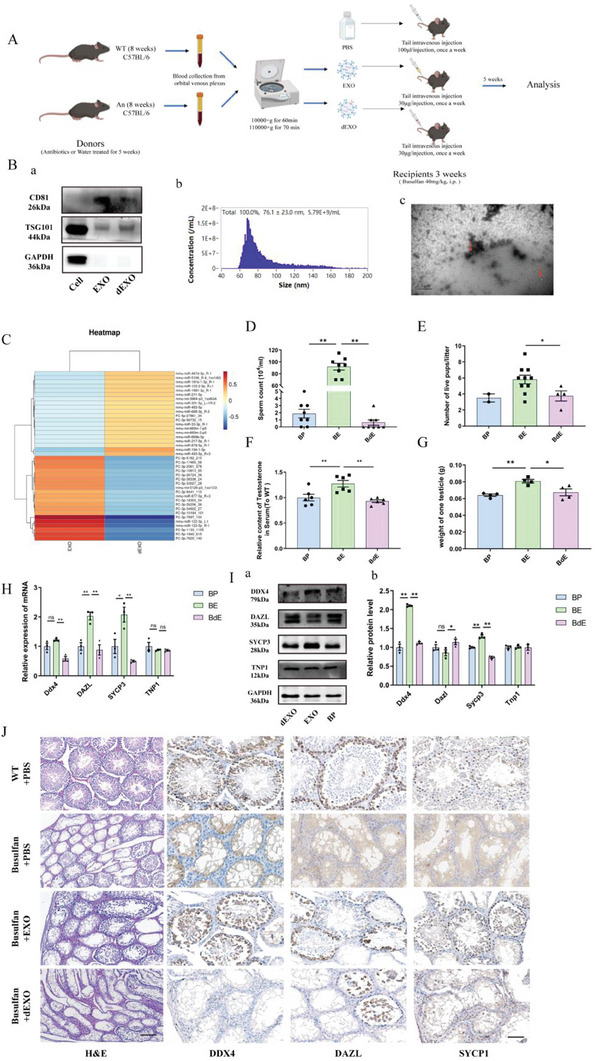
Effects of circulating exosomes from different treatments on spermatogenesis in mice. (Data are expressed as means ± SEM.). A) Experimental scheme. B) Characterization of the exosomes. a) Western blot analysis of protein markers. b) Particle size analysis. c) The morphology of exosomes was observed using transmission electron microscopy. C) Heatmap representation of the relative miRNA differences between WT_serum exosomes (EXO) and An_serum exosomes (dEXO). D) Statistical analysis of sperm count (*n* = 8). E) Statistical analysis of litter size (*n* = 10). F) ELISA analysis of serum testosterone levels in the different treatment groups (*n* = 6). G) Statistical analysis of unilateral testis weight in mice (*n* = 4). H) mRNA quantification using qPCR in the testes (*n* = 3). I) Western blotting a) and quantification b) analysis of proteins. J) Representative testicular sections from the different treatment groups with H&E and Immunohistochemistry (IHC) staining. H&E scale bar = 200 µm; IHC scale bar = 100 µm.

Exosomes, as cytokines, have been shown to play important roles in various biological processes. We hypothesized that the circulating exosomes may mediate spermatogenesis impairment caused by DSGM. To clarify the role of circulating exosomes in the gut‐exosomes‐testes axis, the serum exosomes were isolated and collected from WT mice (EXO) and DSGM mice (dEXO) by differential centrifugation. A mouse model was established by tail vein injection of EXO/dEXO (**Figure** **3**A). The purity and quality of exosomes were determined using western blotting, transmission electron microscopy, and nanoflow. The results showed that the collected samples were consistent with the basic characteristics of exosomes (Figure 3B). The small RNA sequencing analysis showed that the abundance of microRNAs in EXO and dEXO was significantly different (Figure S3A,B, Supporting Information). A heatmap was created to show the top twenty differential microRNAs (Figure 3C). These results indicated that DSGM affects the abundance of microRNAs in exosomes.

Exosome staining was performed to characterize the transport and targeting of exosomes in vivo. CD63 and PKH26 labeling showed that the exosomes reached the testicular tissue of mice through blood circulation and entered the seminiferous tubules (Figures S3C and S10A, Supporting Information). After 5 weeks of exosome treatment, no significant difference was observed in the weight of mice between the busulfan+EXO (BE), busulfan+PBS (BP), and busulfan+dEXO treatment groups (BdE) (Figure S3D, Supporting Information). Compared with the BP and BdE groups, the weight of unilateral testes, the number of sperm in the epididymides, the levels of testosterone in blood serum, the pregnancy rate of female mice, and the litter sizes increased significantly in the BE group (Figure 3D–G). Consistent with the previous results (Figure 1D–I), the number of germ cells in the BE group was significantly increased compared with the BP and BdE groups (Figure 3H,I). The H&E and IHC staining of testicular tissue sections further validated these results (Figure 3J). The above results indicated that exosomes from wild‐type mice alleviated the spermatogenesis impairment induced by busulfan, and the DSGM led to changes in the composition of circulating exosomes, impairing spermatogenesis in mice.

### 2.4 Single‐Cell Transcriptome Sequencing of Testicular Tissue Reveals the Molecular Mechanism of the Effects of Exosomes on Spermatogenesis

**Figure 4:**
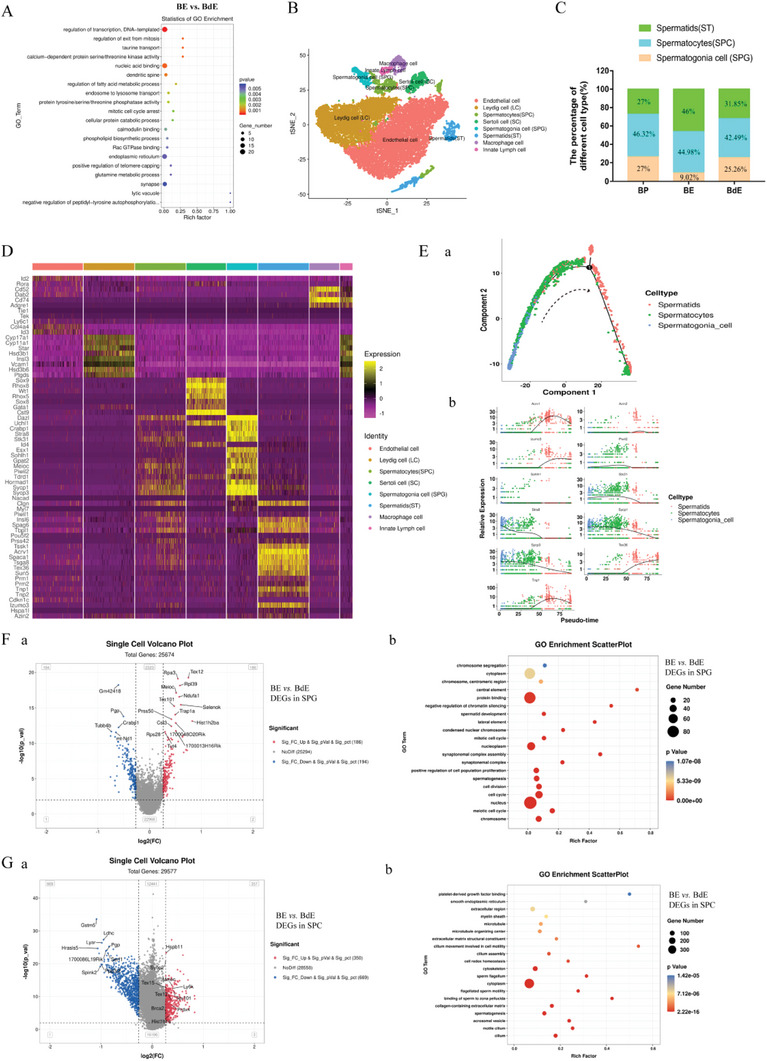
Single‐cell transcriptome sequencing of testicular tissue in different treatment groups. A) GO analysis of DEGs between BE and BdE groups. The top 20 GO biological pathways are shown based on the FDR p‐values. B) tSNE (*t*‐distributed Stochastic Neighbor Embedding) plot of germ cell clusters in testes defined by scRNA‐seq analysis. C) Percentage of each germ cell cluster in the three groups. D) Heatmap of the average expression of marker genes in all cells of each cell population. E) a) Single‐cell trajectories of germ cell subsets are shown with cells ordered in pseudo‐time. b) DEGs among different cell types are shown in the cell tree structural trajectory. F) Volcanic map analysis a) and GO analysis b) of the DEGs in the SPG cluster between the BE and BdE groups. The top 20 GO biological pathways are shown based on the FDR *p*‐values. G) Volcanic map analysis a) and GO analysis b) of DEGs in the SPC cluster between the BE and BdE groups. The top 20 GO biological pathways are shown based on the FDR *p*‐values. The threshold defined as a significantly different species is Fold Change >1.2, *p* < 0.05 or Fold Change < 0.83, *p* < 0.05.

Gene Ontology (GO) enrichment analysis of transcriptome differentially expressed genes (DEGs) in the testes of BE and BdE treatment groups was performed. The results showed that EXO and dEXO significantly differed in the regulation of spermatogenesis, and DEGs were significantly enriched in GO terms such as “regulation of exit mitosis” and “mitosis cell cycle arrest” (**Figure** **4**A), which play important roles in spermatogenesis.

To determine which cell subtypes participate in the regulation of spermatogenesis by exosomes, 10× single‐cell transcriptome sequencing (scRNA‐seq) on the testes of BP, BE and BdE groups was performed. Single‐cell data analysis was performed using OmicStudio tools (https://www.omicstudio.cn/tool). Corresponding to the known cell type marker genes in the testes (Figure S4, Supporting Information), 16 cell clusters were defined as 8 cell types (Figure 4B,D; Figure S5A,B, Supporting Information), including endothelial cells, inner Lynch cells, macrophages, Sertoli cells, Leydig cells (LC), spermatogonia cells (SPG), spermatocyte cells (SPC) and spermatid cells (ST). Germ cells from the BP, BE and BdE groups were used to calculate the percentage of each cell subtype. As shown in Figure 4C, the percentage of SPG clusters (BP: 27%, BE: 9.02%, BdE: 25.26%), SPC clusters (BP: 46.32%, BE: 44.98%, BdE: 42.49%) and ST clusters (BP: 27%, BE: 46%, BdE: 31.85%) differed significantly among the three treatment groups. Pseudo‐time analysis showed that the developmental order of the germ cell subsets was SPG, SPC, and ST (Figure 4E; Figure S5C,D, Supporting Information). The expression profile of marker genes in these cell clusters is shown in Figure 4E. These results indicated that the changes in circulating exosomes induced by DSGM blocked the differentiation of germ cells from spermatogonia to sperm cells.

GO enrichment analysis results showed that, compared with the BP and BdE groups, the DEGs of the BE group in SPG and SPC clusters were significantly enriched in GO terms such as “spermatogenesis,” “spermatid development,” “mitotic cell cycle,” “synaptonemal complex assembly,” “meiotic cell cycle,” “sperm flagellum” and “flagellated sperm motility” (Figure 4F,G; Figure S5E–H, Supporting Information). These results suggested that the changes in circulating exosomes induced by DSGM significantly altered the meiotic process of spermatogenesis.

To assess this hypothesis, the mRNA expression levels of the parts of the representative genes related to meiosis were detected. The results showed that the relative expression of meiosis‐related genes in the An, An_FMT, and BdE groups decreased by varying degrees (Figure S6, Supporting Information). These results indicated that meiosis was inhibited by the changes in circulating exosomes induced by DSGM, thus impairing spermatogenesis by inhibiting meiosis.

### 2.5 Increased miR‐211‐5p in Circulating Exosomes Targets Meioc to Impair Meiosis

To clarify the molecular mechanism of exosomes regulating meiosis, TargetScan Release 7.1 and miRDB online website (https://www.targetscan.org/vert_71/; https://mirdb.org/) were used to predict the target genes of differential miRNAs in EXO and dEXO, and the predicted target genes were cross‐analysed with the DEGs from the scRNA‐seq results. The results showed that there was a targeting relationship between significantly overexpressed miR‐211‐5p in dEXO and Meioc, which is downregulated in the BdE group in both SPG and SPC clusters. Double luciferase reporter gene detection system results further showed that miR‐211‐5p had a strong targeting relationship with Meioc (Figure S7, Supporting Information).

**Figure 5:**
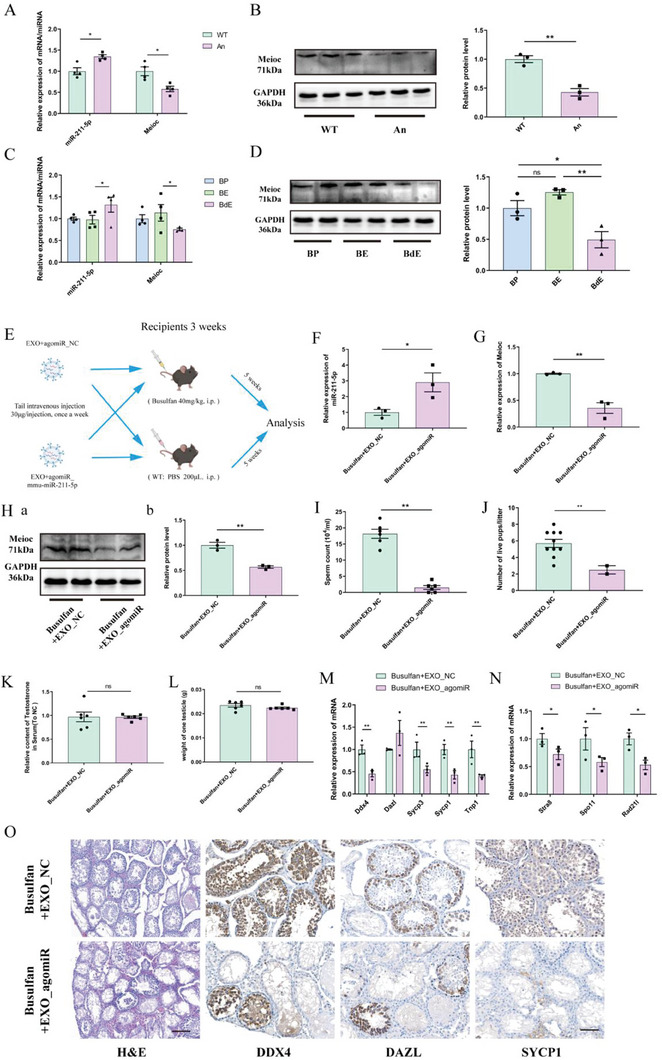
Increased miR‐211‐5p in circulating exosomes targets Meioc to impair meiosis (Data are expressed as means ± SEM.). A) mRNA/miRNA quantification by qPCR in the testes from WT/An groups (*n* = 4). B) Western blotting (left) and quantification (right) analysis of proteins in the testes from WT/An groups (*n* = 3). C) mRNA/miRNA quantification using qPCR in the testes from BP, BE, and BdE groups (*n* = 4). D) Western blotting (left) and quantification (right) analysis of proteins in the testes from BP, BE, and BdE groups (*n* = 3). E) Experimental scheme. F–G) miRNA/mRNA quantification using qPCR in the testes from the busulfan+EXO_NC and the busulfan+EXO_ agomiR groups (*n* = 3). H) Western blotting a) and quantification b) analysis of MEIOC protein in the testes from busulfan+EXO_NC/agomiR groups (*n* = 3). I) Statistical analysis of sperm counts in epididymis from busulfan+EXO_NC/agomiR groups (*n* = 6). J) Statistical analysis of litter size in busulfan+EXO_NC/agomiR groups (*n* = 10). K) ELISA analysis of serum testosterone levels in busulfan+EXO_NC/agomiR groups (*n* = 6). L) Statistical analysis of the unilateral testes weight in busulfan+EXO_NC/agomiR groups (*n* = 6). M) Quantification of mRNA using qPCR in the busulfan+EXO_NC/agomiR mice testes (*n* = 4). N) Quantification of meiosis‐related mRNA expression levels using qPCR in the busulfan+EXO_NC/agomiR group (*n* = 3). O) Representative testicular sections from different treatment groups of busulfan+EXO_NC/agomiR mice with H&E and immunohistochemistry (IHC) staining. H&E scale bar = 200 µm; IHC scale bar = 100 µm.

Studies have shown that MEIOC helps prevent the degradation of meiotic transcripts and interacts with the YTH domain containing 2 to maintain meiotic progress.^[^ ^16^ ^]^ The gene and protein expression levels of miR‐211‐5p and Meioc in the testes of each group were identified. Compared with the WT group, the expression level of miR‐211‐5p in the testes of the An group increased significantly, whilst the mRNA and protein expression level of Meioc decreased significantly. The mice that received FMT from antibiotic‐treated mice showed consistent changes in mRNA and protein expression levels (**Figure** **5**A,B; Figure S8A–D, Supporting Information). The changes of miRNA and Meioc in the testes of the BP, BE, and BdE groups were consistent with the previous results. Compared with the BE group, the expression level of miR‐211‐5p in the testes of the BdE group increased, and the mRNA and protein levels of Meioc decreased significantly (Figure 5C,D). This conclusion was also verified in vitro (Figure S8E–H, Supporting Information). With overexpression of miR‐211‐5p in the GC1 and GC2 cell lines, Meioc expression decreased significantly (Figure S8I–L, Supporting Information). Importantly, like antibiotic treatment, the level of miR‐211‐5p in the circulating exosomes of An_FMT mice was significantly increased compared to the WT_FMT group (Figure S9A,B, Supporting Information). Altogether, the results indicated that DSGM increased the expression of miR‐211‐5p in the circulating exosomes and inhibited the expression of Meioc in testes.

To define the key role of exosomal miR‐211‐5p in mouse spermatogenesis, exosomes from WT mice transfected with agomiR_mmu‐miR‐211‐5p were used to simulate the circulating exosomes of mice with DSGM (Figure 5E). After 5 weeks of treatment, the change in miR‐211‐5p expression in the circulating exosomes of each group was ascertained, and the results showed a significant increase in miR‐211‐5p expression in the EXO_agomiR groups (Figure S9C,D, Supporting Information). Quantitative results showed that compared with the EXO_NC group, the expression of miR‐211‐5p in the EXO_agomiR group was significantly increased, and the mRNA and protein expression levels of Meioc in the testicular tissue of the EXO_agomiR group decreased significantly (Figure 5F–H; Figure S9E,F, Supporting Information).

Moreover, compared with the EXO_NC group, the number of sperm in the epididymides, the pregnancy rate of the female mice, and the litter sizes in the EXO_agomiR group decreased significantly, but there was no significant difference in testicular weights and testosterone levels in the serum (Figure 5I–L; Figure S9G–J, Supporting Information). The results also showed that, compared with the EXO_NC group, the expression level of marker genes of total germ cells, spermatocytes, and spermatids in the EXO_agomiR group decreased significantly (Figure 5M; Figure S9K, Supporting Information). Additionally, the mRNA expression level of representative genes related to meiosis in the EXO_agomiR group decreased significantly (Figure 5N; Figure S9L, Supporting Information). H&E and IHC staining of testicular tissue sections showed that compared with the EXO_NC group, the structure of convoluted seminiferous tubules in the EXO_agomiR group was damaged, the number of convoluted seminiferous tubules containing germ cells decreased significantly, and the number of DDX4^+^, DAZL^+^ and SYCP1^+^ cells in convoluted seminiferous tubules decreased significantly (Figure 5O; Figure S9M, Supporting Information), meaning that abnormal elevation of miR‐211‐5p in circulating exosomes impairs spermatogenesis.

**Figure 6:**
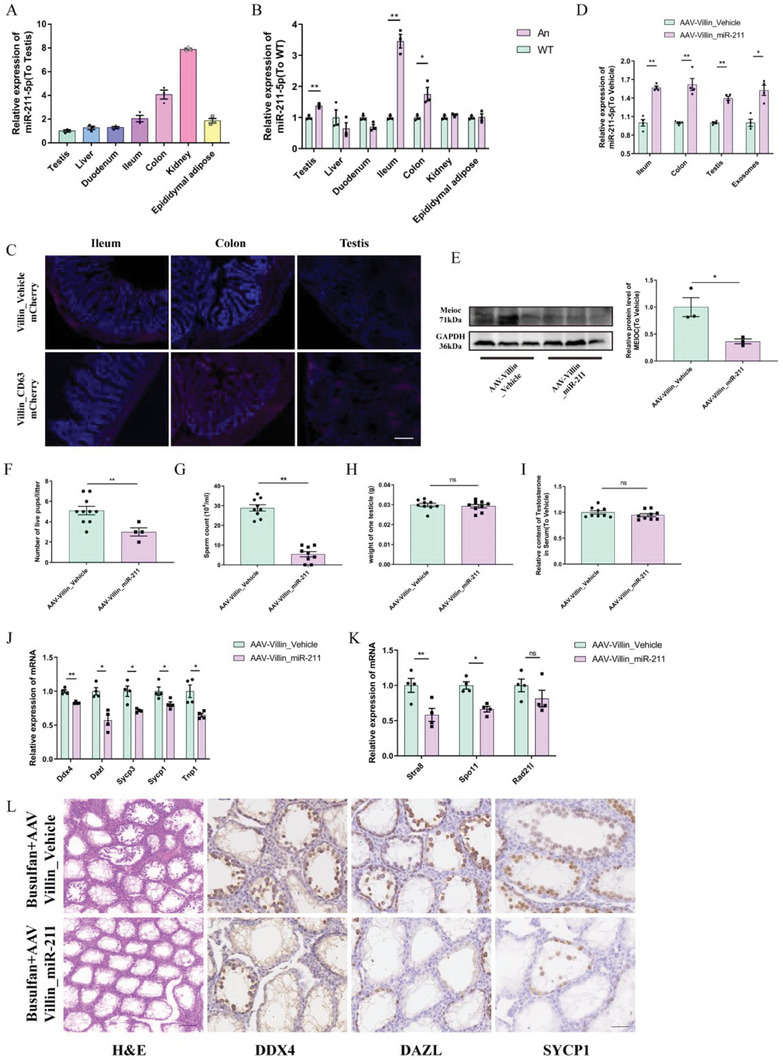
Gut‐derived abnormal elevation of miR‐211‐5p impairs spermatogenesis (Data are expressed as means ± SEM.). A) Relative expression levels of miR‐211‐5p from major tissues and organs of WT mice (*n* = 3). B) Relative expression levels of miR‐211‐5p from major tissues and organs in WT/An groups (*n* = 3). C) Representative frozen tissue sections of different tissues from different treatment groups. Scale bar = 50 µm. (Villin_mCherry, Villin promoter, and mCherry fluorescent protein, fluorescence is only detected in intestinal tissue; Villin_CD63 mCherry, Villin promoter and mCherry fluorescent labeled CD63 protein, fluorescence was detected in tissues of the intestine, testis, and liver). D) miRNA quantification using qPCR in the testes from the busulfan+AAV‐Villin_Vehicle/miR‐211 groups (*n* = 4). E) Western blotting (left) and quantification (right) analysis of MEIOC protein in the testes from busulfan+AAV‐Villin_Vehicle/miR‐211 groups (*n* = 3). F) Statistical analysis of litter size in busulfan+AAV‐Villin_Vehicle/miR‐211 groups (n = 10). G) Statistical analysis of sperm counts in epididymis from busulfan+AAV‐Villin_Vehicle/miR‐211 groups (*n* = 9). H) Statistical analysis of the unilateral testes weight in busulfan+AAV‐Villin_Vehicle/miR‐211 groups (*n* = 9). I) ELISA analysis of serum testosterone levels in busulfan+AAV‐Villin_Vehicle/miR‐211 groups (*n* = 9). J) Quantification of mRNA using qPCR in busulfan+AAV‐Villin_Vehicle/miR‐211 groups (*n* = 4). K) Quantification of meiosis‐related mRNA expression levels using qPCR in busulfan+AAV‐Villin_Vehicle/miR‐211 groups (*n* = 4). L) Representative testicular sections from different treatment groups of busulfan+AAV‐Villin_Vehicle/miR‐211 groups mice with H&E and immunohistochemistry (IHC) staining. H&E scale bar = 200 µm; IHC scale bar = 100 µm.

These results indicated that abnormal up‐regulation of miR‐211‐5p in circulating exosomes inhibits meiosis and impairs spermatogenesis by inhibiting Meioc expression in the testes.

### 2.6 Abnormal Elevation of miR‐211‐5p in Gut‐Derived Circulating Exosomes Impairs Spermatogenesis

The above results showed that miR‐211‐5p plays a significant role in impairing mouse spermatogenesis induced by DSGM, but its source is still unclear. Therefore, the present study evaluated the relative expression abundance of miR‐211‐5p in mouse liver, duodenal, ileal, colon, kidney, epididymal adipose, and testicular tissues, as well as the differential changes caused by dysbiosis of gut microbiota. The results showed that the relative abundance of miR‐211‐5p was highest in the kidneys of mice, followed by the intestinal tissue (**Figure** **6**A). Interestingly, compared with the WT group mice, the expression levels of miR‐211‐5p in the testes, ileum, and colon tissues of the An group were significantly increased (Figure 6B). It is reasonable to hypothesize that the main source of abnormally elevated miR‐211‐5p in the testes may be intestinal tissue.

To verify this hypothesis, a Lentivirus vector (LV Villin‐CD63‐mCherry) specifically expressing CD63‐mCherry in the intestine was constructed. The results showed that the constructed vector was specifically expressed in the intestinal tissue while the CMV promoter vector was also expressed in the liver and testicular Leydig (Figure S10A, Supporting Information), and the exosomes with mCherry‐labelling CD63 from the intestine reached the inside of the seminiferous tubules by crossing the blood‐testis barrier (Figure 6C). Further experimental results showed that the adeno‐associated virus vector (AAV‐Villin_miR‐211) with intestinal‐specific overexpression of pre‐miR‐211‐5p was specifically expressed in intestinal tissue while the CMV promoter vector is also expressed in the liver, adipose, and testicular Leydig (Figure S10B, Supporting Information).

The results showed that compared with the AAV‐Villin_vehicle group, the relative abundance of miR‐211‐5p in the ileum, colon, testicular tissues, and circulating exosomes was significantly increased in the AAV‐Villin_miR‐211 group mice (Figure 6D; Figure S11A, Supporting Information), while the protein expression level of MEIOC in testicular tissues was significantly reduced (Figure 6E; Figure S11B, Supporting Information).

Compared with the AAV‐Villin_vehicle group, the number of sperm in the epididymides of male mice, the pregnancy rate of female mice, and the litter size in the AAV‐Villin_miR‐211 group decreased significantly, but there was no significant difference in testicular weights and serum testosterone levels (Figure 6F–I; Figure S11C–F, Supporting Information). Furthermore, compared with the AAV‐Villin_vehicle group, the expression level of marker genes of total germ cells, spermatocytes, and spermatids in the AAV‐Villin_miR‐211 group decreased significantly. Similarly, the mRNA expression levels of representative genes related to meiosis in the AAV‐Villin_miR‐211 group decreased significantly (Figure 6J–K; Figure S11G,H, Supporting Information). H&E and IHC staining of testicular tissue sections showed that intestinal‐specific overexpression of miR‐211‐5p caused severe damage to the convoluted seminiferous tubules in mice, which mainly manifested in a significant reduction of the number of germ cells and the destruction of the seminiferous epithelium cycle of convoluted seminiferous tubules (Figure 6L; Figure S11I, Supporting Information). These results indicated that the abnormal elevation of miR‐211‐5p in gut‐derived circulating exosomes impairs meiosis and spermatogenesis by inhibiting Meioc expression in the testes.

## 3 Discussion

Previous studies have demonstrated that intestinal microbiota plays an important role in regulating the immune homeostasis of the host intestinal epithelium,^[^ ^17^ ^]^ the occurrence and development of metabolic diseases,^[^ ^18^ ^]^ the occurrence and development of cancer,^[^ ^19^ ^]^ learning and cognitive functions^[^ ^20^ ^]^ and fertility.^[^ ^6^, ^21^ ^]^ Here, we confirmed that the DSGM impaired the spermatogenesis and fertility of mice, while the transplantation of fecal microbiota from healthy mice alleviated the reproductive damage (Figures 1 and 2; Figure S2, Supporting Information). Notably, the litter size of female mice mated with the antibiotic treatment group significantly decreased, but the pregnancy rate did not change. The mechanism needs investigation to determine whether, for example, the fertilization ability of sperm had changed or epigenetic changes had taken place in the sperm, resulting in a change in the developmental competence of embryos. In addition, changes in testosterone will also be an interesting phenomenon that requires further in‐depth and detailed research in the future. Although DSGM regulates spermatogenesis by regulating the metabolism of vitamin A and sphingosine,^[^ ^21^, ^22^ ^]^ other regulatory mechanisms or regulatory molecules of the “gut‐testes axis” still need to be explored. We provided new insights into the mechanism through which gut‐derived circulating exosomes act as molecular mediators of host reproductive damage induced by gut microbiota dysbiosis.

Exosomes are molecular mediators transported by blood circulation, entering distal recipient cells through endocytosis.^[^ ^11^ ^]^ Studies have shown that HFD induces the transformation from phosphatidylethanolamine to phosphatidylcholine in exosomes and increases microbiota derivative DNA to regulate insulin sensitivity.^[^ ^13^, ^23^ ^]^ Additionally, miR‐9‐3p in the extracellular vesicles derived from adipose tissue targets and inhibits the cognitive impairment caused by BDNF‐induced insulin resistance.^[^ ^23^ ^]^ In men, the extracellular vesicles in semen are related to post‐testicular sperm maturation, including the acquisition of sperm vitality and the reduction of oxidative stress.^[^ ^14^, ^24^ ^]^ However, the function of circulating exosomes in reproduction has not been confirmed by conclusive experimental evidence. Here, we proved for the first time that the exosomes from a non‐reproductive system can reach the seminiferous tubules of the testes and regulate spermatogenesis.

Most functions of exosomes are attributed to miRNAs, which are single‐stranded noncoding RNAs composed of 19 to 22 nucleotides.^[^ ^25^ ^]^ A recent study showed that DSGM led to changes in the composition of plasma exosomes.^[^ ^26^ ^]^ Our results showed that the gut‐derived circulating exosomes from DSGM mice impaired spermatogenesis, while the exosomes from the WT mice saved the spermatogenesis damage induced by busulfan (Figures 3 and 4; Figure S6, Supporting Information). The scRNA‐seq results showed that the circulating exosomes from DSMG mice blocked spermatogenesis in mice by damaging the meiosis process. Further analysis confirmed that miR‐211‐5p in exosomes damages the meiotic process by inhibiting Meioc. Studies have shown that Meioc plays an important role in preventing the degradation of meiotic transcripts and interacting with YTHDC2 to maintain meiotic progress.^[^ ^16^, ^27^ ^]^ In addition, studies have reported that Meioc plays an important role in regulating the transition from mitosis to meiosis, as well as the m6A transcripts during spermatogenesis.^[^ ^16^, ^27^ ^]^ Here we identified miR‐211‐5p as a key factor, which was carried by circulating exosomes to seminiferous tubules to induce spermatogenesis impairment and fertility damage in mice with DSGM by targeting Meioc. miRNA sequencing revealed that miR‐211‐5p was significantly up‐regulated in the gut‐derived circulating exosomes and testicular tissues of mice with DSGM, indicating that miR‐211‐5p acts as a powerful regulator, leading to spermatogenesis impairment. Consistent with our results, a recent study showed that miR‐204‐5p, which has a highly similar sequence to miR‐211‐5p, is significantly up‐regulated in circulating exosomes of diabetes patients.^[^ ^23^ ^]^ Besides, the online database of EVmiRNA (http://bioinfo.life.hust.edu.cn/EVmiRNA#!/) showed that miR‐211‐5p was highly expressed in the colon and highly enriched in the exosomes of colon cancer patients. Clinical studies have shown that in patients with congenital intestinal atresia and Crohn's disease, the expression of miR‐211‐5p in the intestinal mucosa is significantly up‐regulated and accompanied by DSGM.^[^ ^28^ ^]^ The incidence of sexual dysfunction in patients with inflammatory bowel disease is 15–25% in men.^[^ ^29^ ^]^ Our results showed that DSGM disturbs normal spermatogenesis by affecting the level of miR‐211‐5p loaded in the gut‐derived circulating exosomes (Figures 5 and 6; Figure S10 and S11, Supporting Information).

Although this work has proposed new insights into the “gut‐exosome‐testis” axis and established a clear relationship between intestinal microbiota and circulating exosomes, the exact signaling mechanism that mediates changes in exosome cargo loading remains unclear. Future work may determine how intestinal microbiota regulate miRNA expression and affect circulating exosome cargo loading.

## 4 Conclusion

**Figure 7:**
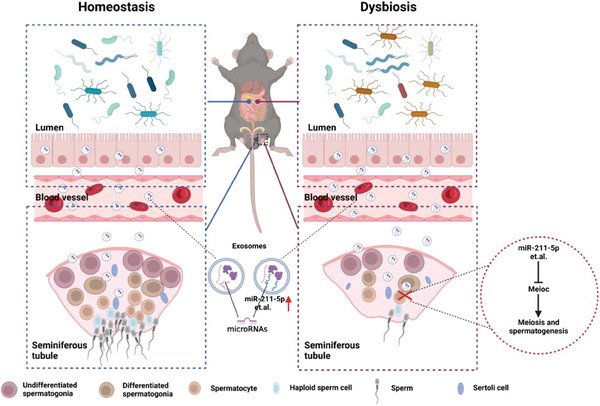
Conclusion. Dysbiosis of gut microbiota led to the abnormal upregulation of miR‐211‐5p in gut‐derived circulating exosomes, which regulates the meiosis, specifically targeting the coiled‐coil domain (Meioc) in the testes, thus impairing spermatogenesis.

We identified a new mechanism of DSGM‐induced reproductive dysfunction in male mice. Following DSGM, miR‐211‐5p loaded in the gut‐derived circulating exosomes was increased abnormally and transported to the testicular seminiferous tubules through the blood circulation, targeting and inhibiting the expression of Meioc, consequently impairing spermatogenesis(**Figure** **7**). FMT or engineered exosomes loaded with drugs may provide alternative strategies for drug intervention to treat spermatogenesis impairment induced by DSGM. Additionally, the level of miR‐211‐5p in the circulatory system may serve as a predictive indicator for male reproductive injury.

## 5 Experimental Section

### Resource Availability

Further information and requests for resources and reagents should be directed to and will be fulfilled by the corresponding author Xu Zhou (xzhou65@vip.sina.com).

### Materials Availability

This study did not generate new reagents.

### Animals and Tissue Sampling

Animals were handled according to protocols approved by the Animal Ethics Association of Jilin University (Approval number: SY202105021). Wild‐type C57BL/6 mice were purchased from Changsheng Biotechnology Co. Ltd., Liaoning, China. All animals were raised in the specific pathogen‐free animal lab of Jilin University (25 ± 2 °C; relative humidity, 45–60%; light: dark cycle, 12:12 h). During the experiment, all animals had free access to food and drinking water.

Samples required for single‐cell‐RNA‐seq and 16S rDNA‐seq were collected, snap‐frozen, and stored at −80 °C for analysis.

### Antibiotic Treatment

Mice were administered a mixture of antibiotics (Vancomycin 0.5 mg mL^−1^, Neomycin 1 mg mL^−1^, Ampicillin 1 mg mL^−1^, Metronidazole 1 mg mL^−1^; Shanghai yuanye Bio‐Technology Co., Ltd) in drinking water for four weeks according to established protocols.^[^ ^30^ ^]^

### Fecal Microbiota Transplantation

Fecal samples were diluted in sterile saline to a working concentration of 0.05 g mL^−1^ and filtered through a 70‐µm cell strainer. Fresh transplant material was prepared within 10 min before oral gavage to prevent changes in bacterial composition. Oral gavage with 200 µL fecal transplant material was conducted daily throughout the 5‐week experiment according to established protocols.^[^ ^6^, ^22^ ^]^

### In Vivo Exosome Treatment

Exosomes isolated from serum were transferred into recipient mice via tail vein injection. Mice received 30 µg per injection, once per week, as a previous study reported that the quantity of circulating extracellular vesicles in obese mice was ≈30 µg per mouse.^[^ ^23^ ^]^

### Cell Culture and Exosome Treatment

The GC1_SPG (Procell Life Science & Technology Co., Ltd, China) cell line was cultured with DMEM/F12 (Sigma–Aldrich, USA) supplemented with 10% FBS without exosomes (ABW, Uruguay) and 1% Penn/Strep. The GC2_SPC (Procell Life Science&Technology Co., Ltd, China) cell line was cultured with DMEM/High Glucose (Sigma–Aldrich, USA) supplemented with 10% FBS without exosomes (ABW, Uruguay) and 1% Penn/Strep. For in vitro exosomes treatment, 5 µg of exosomes was added to 1 × 105 cultured cells. For in vitro miR_mimic/NC treatment, 50 × 10–6 m of miR_mimic/NC was added to cultured cells.

### Extraction and Purification of miRNA from Exosomes/Tissue

According to the manufacturer's protocol, serum exosome/tissue miRNA was isolated and purified using the miRNeasy Serum/Plasma Kit (QIAGEN, Cat. No. 217 184). The content determination and peak value in the control were used for standardization based on the same amount of Spike‐in RNA (QIAGEN, Cat. No. 339 390).

### Exosome Isolation and Characterisation

The serum was collected according to protocols approved by the Animal Ethics Association of Jilin University. Briefly, the blood was collected and left at room temperature for 30 min, centrifuged at 4500 × g for 15 min, and the supernatant was collected and centrifuged at 10 000 × g for 60 min to remove large vesicles. Then, the supernatant was centrifuged at 110 000 × g for 90 min. The exosomes were resuspended in PBS and filtered through a 0.22‐µm filter. Exosomes from different mice were collected and stored at −80 °C for further experiments.

For the characterization of exosomes, particle size analysis was performed using a NanoSight instrument (Flow Nano Analyzer, Xiamen, China). The morphology of exosomes was observed via transmission electron microscopy, and the protein content of exosomes was determined using western blotting.

### PKH26 Labeling and Exosome Trafficking

To monitor exosome trafficking, exosomes were labeled with PKH26 fluorescent dye using a PKH26 fluorescence cell linker kit (Sigma–Aldrich, USA). After staining, the exosomes were washed with PBS and collected via ultracentrifugation (100 000 × g for 60 min) at 4 °C. Finally, PKH26‐labeled exosomes were resuspended in PBS and transferred into recipient mice via tail vein injection. The mouse testes and liver were obtained 48 h after PKH26‐labeled exosome injection.

### Lentivirus and Adeno‐Associated Virus Vectors

LV‐CD63‐mCherry‐WPRE and AAV‐microRNA‐CMV‐EGFP‐WPRE‐hGH_polyA vectors were provided by Wuhan BrainVTA Science and Technology Co., Ltd. Lentivirus was administered by intraperitoneal injection with a dosage of 2 × 108 Tu; Adenovirus was administered via intraperitoneal injection at a dosage of 5 × 1010 v.g, injected every three weeks.

### Overexpression of microRNA in Exosomes

To verify the function of microRNAs (miRNAs) in exosomes, the exosomes were incubated with mmu‐miR‐211‐5p agomiR/agoNC (5 nmol/30 µg) (Guangzhou RiboBio Co., Ltd.) at room temperature for 10 min. After incubation, the exosomes were washed with PBS and collected via ultracentrifugation (110 000 × g, 4 °C, 90 min). Finally, exosomes_agomiR/NC (EXO_agomiR/EXO_NC) was resuspended with PBS and stored at −80 °C for subsequent experiments.

### Morphology and Immunohistochemistry

Testicular tissue was promptly collected, fixed in a 4% formaldehyde solution, dehydrated, and embedded in paraffin. The thickness of continuous testicular sections was 5 µm. After dewaxing, sections were prepared and stained with hematoxylin and eosin. Three testicular cross‐sections were examined in each group, with at least 30 intervals between cross‐sections.

For immunohistochemistry, the sections were performed in 0.01 m sodium citrate at 95 °C for 10 min. Samples were then blocked with BDT (3% BSA, 10% 29 normal donkey serum in TBS) for 30 min. Antibody source and dilutions were as follows: DDX4 (abcam, ab13840, 1:800), DAZL (abcam, ab34139, 1:800), SYCP3 (abcam, ab97672, 1:400), and SYCP1 (OmnimAb, OM117582, 1:100).

### Sperm Count

The cauda epididymides were dissected from adult mice and incubated at 37 °C for 30 min to release sperm. The sperm were suspended in 1 mL PBS, washed three times, spread on the slide, and fixed for staining. The field of view was captured with a CCD camera. The sperm count was quantified under OLYMPUS optical microscope using a hematology analyzer.

### Parameters of Breeding Rate and Litter Size

At the end of the experimental treatment, male mice were mated with 3‐month‐old female mice. The ratio of male to female mice was 1:2. The vaginal plug of female mice was checked daily until eight days post coitum. When a vaginal plug was found, each female mouse was transferred to a single cage for the evaluation of litter size. The pregnancy rate at eight days post coitum and litter size was calculated.

### The 16S rDNA Sequencing

The total DNA of the microbiome was extracted, and the quality of DNA extraction was detected using agarose gel electrophoresis. At the same time, the DNA was quantified using ultraviolet spectrophotometry. PCR products were purified by AMPure XT beans (Beckman Coulter Genomics, Danvers, MA, USA) and quantified using Qubit (Invitrogen, USA). The amplicon pool was used for sequencing, and the size and quantity of the amplicon library were evaluated using the library quantitative kit of Agilent 2100 biological analyzer (Agilent, USA) and Illumina (Kapa Biosciences, Woburn, MA, USA). Library sorting was performed on the NovaSeq PE250 platform. The samples were sequenced on the Illumina NovaSeq platform according to the manufacturer's instructions and were provided by LC Bio. Bioinformatic analysis was performed using the OmicStudio tools at https://www.omicstudio.cn/tool. The threshold defined as a significantly different species is *p* < 0.05.

### Sequencing of miRNA in Exosomes

The experimental process was carried out according to the standard steps provided by Illumina company, including library preparation and sequencing experiments. The small RNA sequencing library was prepared using the TruSeq Small RNA Sample Prep Kits (Illumina, San Diego, USA) kit. After the library preparation work is completed, the constructed library is sequenced using Illumina Hiseq2000/2500, with a single‐ended reading length of 1× 50 bp. Both the library preparation and sequencing were performed by LC Bio. Bioinformatic analysis was performed using the OmicStudio tools at https://www.omicstudio.cn/tool. The threshold defined as a significantly different species is Fold Change >2, *p* < 0.05 or Fold Change < 0.5, *p* < 0.05.

### Single‐Cell Library Preparation, Sequencing, and Analysis

Single‐cell libraries were prepared using the 10×Genomics Chromium Single Cell 3′Library & Gel Bead Kit v2 37 (10×Genomics Inc, USA) following the manufacturer's instructions. The constructed library was high throughput sequenced using the dual terminal sequencing mode of the Illumina sequencing platform. Both the library preparation and sequencing were performed by LC Bio. Bioinformatic analysis was performed using the OmicStudio tools at https://www.omicstudio.cn/tool. The threshold defined as a significantly different species is Fold Change >1.2, *p* < 0.05 or Fold Change < 0.83, *p* < 0.05.

### Quantitative Real‐Time‐PCR

TRIzol (Takara, Japan) was used to extract total RNA from the tissues according to the manufacturer's instructions. cDNA synthesis was performed using the PrimeScript RT reagent Kit (Takara Bio, Japan). SYBR green‐based qPCR assays were performed with an Agilent Mx3005p (Agilent, Foster City, CA, USA) thermocycler (20 µL assay volume) in a two‐step reaction process. The relative expression levels of mRNAs were calculated by the 2‐∆∆Ct method, with peptidylprolyl isomerase A (Ppia) as the internal reference gene49. The primer sequences are provided in Figure S12 (Supporting Information).

### Western Blot Analysis and Quantification

Cell and tissue lysates were prepared with Radio Immunoprecipitation Assay buffer containing a protease/phosphatase inhibitor (Solarbio Life Sciences). The total protein content was detected using a BCA Protein Assay Kit (Beyotime Biotechnology). The same amount of protein (10–20 µg total) was used for subsequent experiments according to established protocols50. Antibodies were diluted as follows: DDX4 (abcam, ab13840, 1:2000), DAZL (abcam, ab34139, 1:2000), SYCP3 (abcam, ab97672, 1:2000), SYCP1 (OmnimAb, OM117582, 1:1000), TNP1 (ThermoFisher, PA5‐100119, 1:1000), MEIOC(ORIGENE, TA334967, 1:1000).

Finally, the immunoreactions were detected using an enhanced chemiluminescence (ECL) detection kit (Beyotime Biotechnology, China), and images were obtained with a Tanon‐5200 fully automated digital gel imaging analysis system (Shanghai Tanon technology, China).

### Serum Testosterone Measurement

Serum samples were processed, and testosterone concentration was measured using an ELISA kit (Shanghai Langdon Biotechnology Co., Ltd, Shanghai, China). Intra batch differences – CV < 10%. Differences between batches – CV <12%. Sensitivity −0.5 nmol mL^−1^. Detection range: 0.5–120 nmol/mL.

### Statistical Analysis

Data was analyzed and displayed using Prism 8 software (GraphPad). Statistical differences between groups were determined using ANOVA with multiple comparisons using LSD post hoc analysis. Student's *t‐*test was used for comparisons between two parameters. The statistical details for individual experiments can be found in figure legends. The *n*‐value details can also be found in figure legends. Data were expressed as means ± SEM. *p* < 0.05, “*” was considered significant; *p* < 0.01, “**” as very significant; and *p* < 0.001, “***” as highly significant.

### Ethics Approval and Consent to Participate

Animals were handled according to protocols approved by the Animal Ethics Association of Jilin University (Approval number: SY202105021).

## Conflict of Interest

The authors declare no competing interests.

## Author Contributions

X.Z. and C.L. conceived and designed the study. T.C. performed all experiments. M.C., C.S., and G.H. performed exosome isolation and characterization. X.C., K.L., Y.L., C.Y., T.C., N.W., and B.Z. analyzed the data. T.C. wrote the manuscript. X.Z., L.C., and C.L. applied for projects and funding. X.Z. and C.L. supervised the study and edited the manuscript.

## Supporting information

Supporting Information

## Data Availability

The data generated in the study has been uploaded to a public database, and a detailed description of the database URL link is included in the manuscript data availability statement
