# Supplementary material for: Gut‐Derived Exosomes Mediate the Microbiota Dysbiosis‐Induced Spermatogenesis Impairment by Targeting Meioc in Mice
Source: Adv Sci (Weinh). 2024 Mar 25;11(22):2310110. doi: 10.1002/advs.202310110 (PMC11165515; doi:10.1002/advs.202310110)
Supplement: Supplementary file 1 — Supporting Information [file ADVS-11-2310110-s001.pdf]

Supporting Information

for *Adv. Sci.*, DOI 10.1002/advs.202310110

Gut-Derived Exosomes Mediate the Microbiota Dysbiosis-Induced Spermatogenesis Impairment by Targeting Meioc in Mice

Tong Chen, Boqi Zhang, Guitian He, Nan Wang, Maosheng Cao, Caomeihui Shen, Xue Chen, Lu Chen, Kening Liu, Yuxin Luo, Yiqiu huang, Chenfeng Yuan, Xu Zhou and Chunjin Li**

Supporting information

Gut-derived exosomes mediate the microbiota dysbiosis-induced spermatogenesis impairment by targeting Meioc in mice

Tong Chen, Boqi Zhang, Guitian He, Nan Wang, Maosheng Cao, Caomeihui Shen, Xue Chen, Lu Chen, Kening Liu, Yuxin Luo, Yiqiu huang, Chenfeng Yuan, Xu Zhou*, and Chunjin Li*

College of Animal Sciences, Jilin University, Changchun, Jilin, China

***Corresponding author:**

Chunjin Li

College of Animal Sciences, Jilin University, Changchun, Jilin, China

Phone:+8613154399391

Email: llcjj158@163.com

Xu Zhou

College of Animal Sciences, Jilin University, Changchun, Jilin, China

Phone:+8613944099656

Email: xzhou65@vip.sina.com

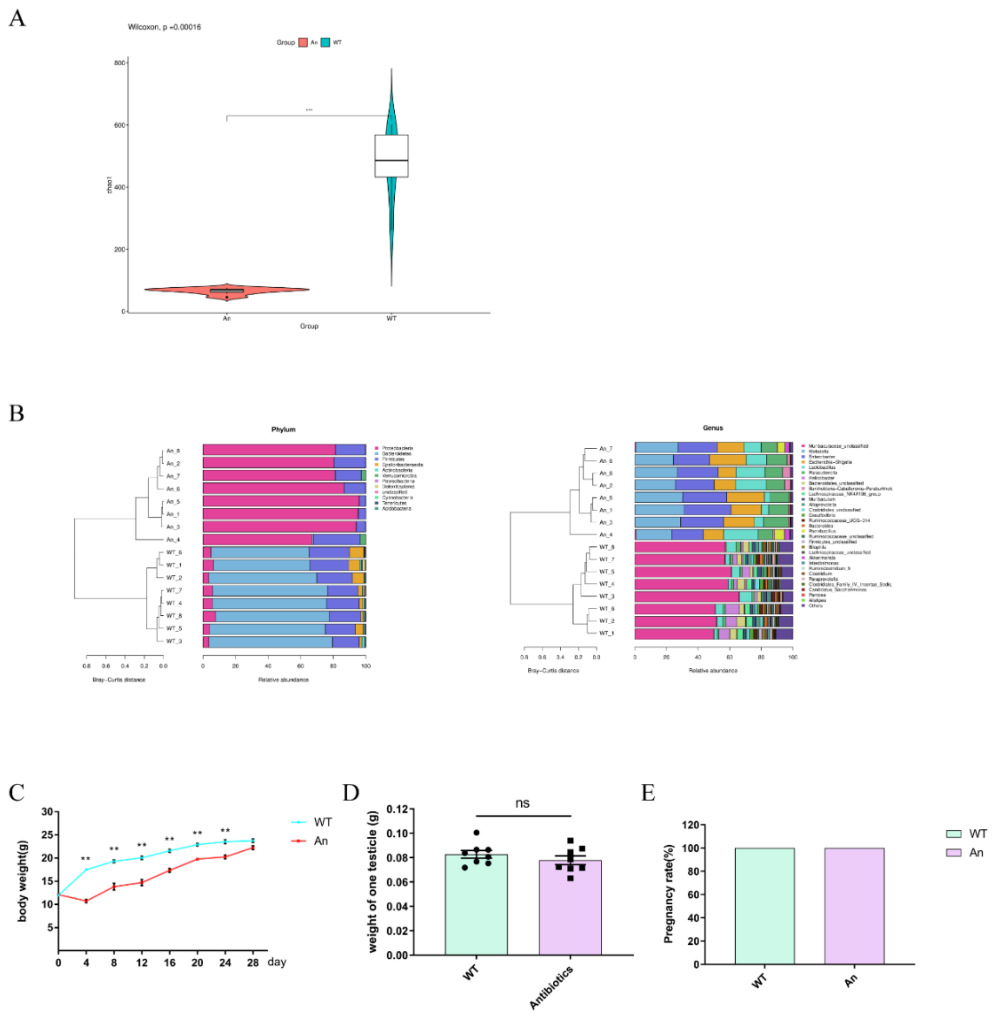

Figure S1. Effect of antibiotic-induced dysbiosis of the gut microbiota on fertility in mice. (Data are expressed as means ± SEM.)

(A) The alpha diversity (species richness) of intestinal microbiota from different treatment groups (n = 8).

(B) Bacterial taxonomic profiling at the phylum (left) and genus (right) levels of gut bacteria from different treatment groups (n = 8).

(C) Statistical analysis of body weight in the different treatment groups (n = 8).

(D) Statistical analysis of unilateral testis weights in the different treatment groups (n = 8).

(E) Statistical analysis of the pregnancy rate in female mice (n = 8).

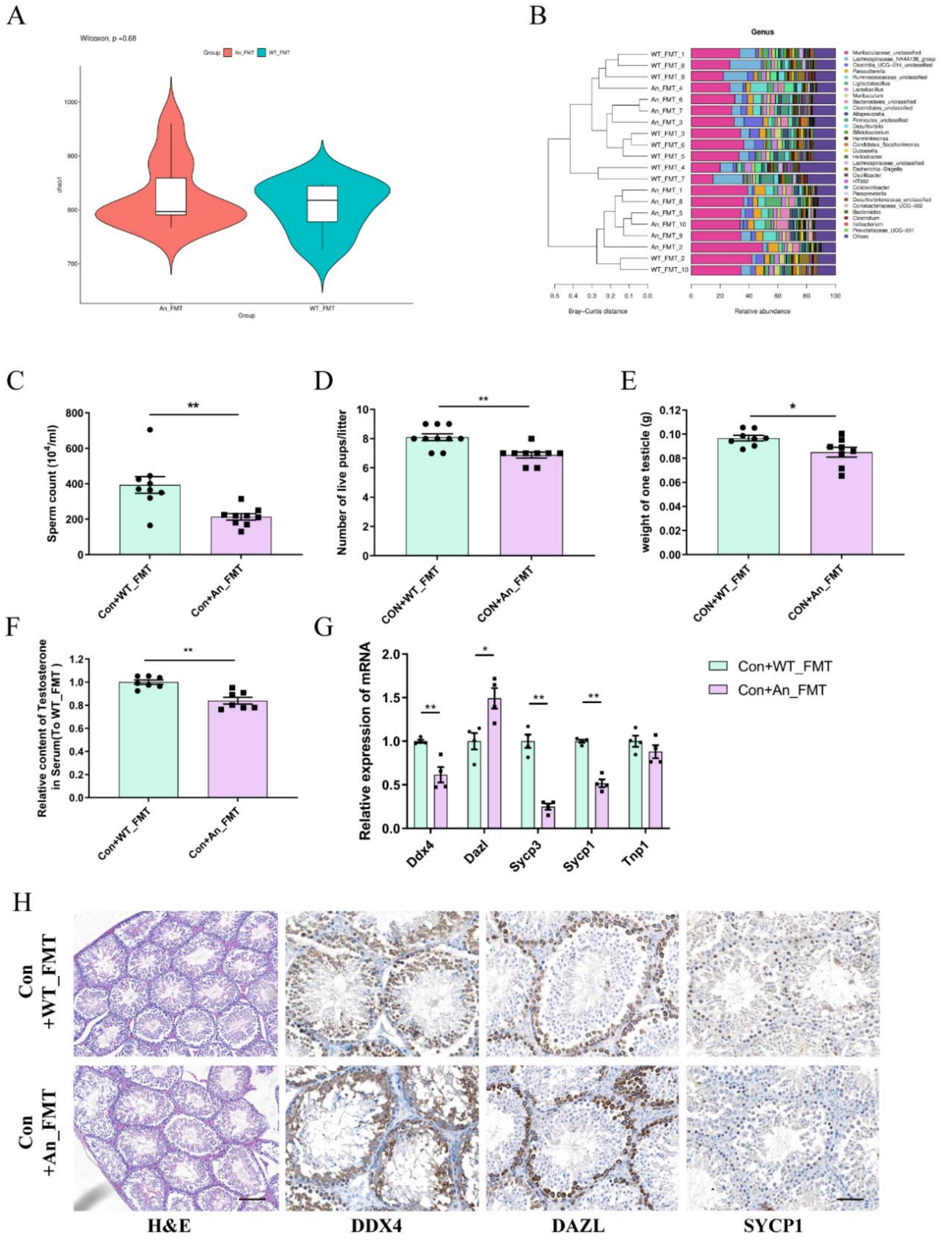

Figure S2. Effect of faecal microbiota transplantation (FMT) on spermatogenesis in wild-type (WT) mice. (Data are expressed as means ± SEM.)

(A) The alpha diversity (species richness) of intestinal microbiota in the receptor mice (n = 10).

(B) Bacterial taxonomic profiling at the genus levels of gut bacteria from the different

treatment groups (n = 10).

(C) Statistical analysis of sperm counts in WT and FMT mice (n = 9).

(D) Statistical analysis of litter sizes in the WT+FMT mice (n = 10).

(E) Statistical analysis of the unilateral testis weights in WT and FMT mice (n = 8).

(F) ELISA analysis of serum testosterone from different treatment groups in WT + FMT mice (n = 7).

(G) Quantification of mRNA by qPCR in the WT + FMT mice testes (n = 4).

(H) Representative testicular sections from different treatment groups of WT + FMT mice with H&E and immunohistochemistry (IHC) staining. H&E scale bar = 200 μm; IHC scale bar = 100 μm.

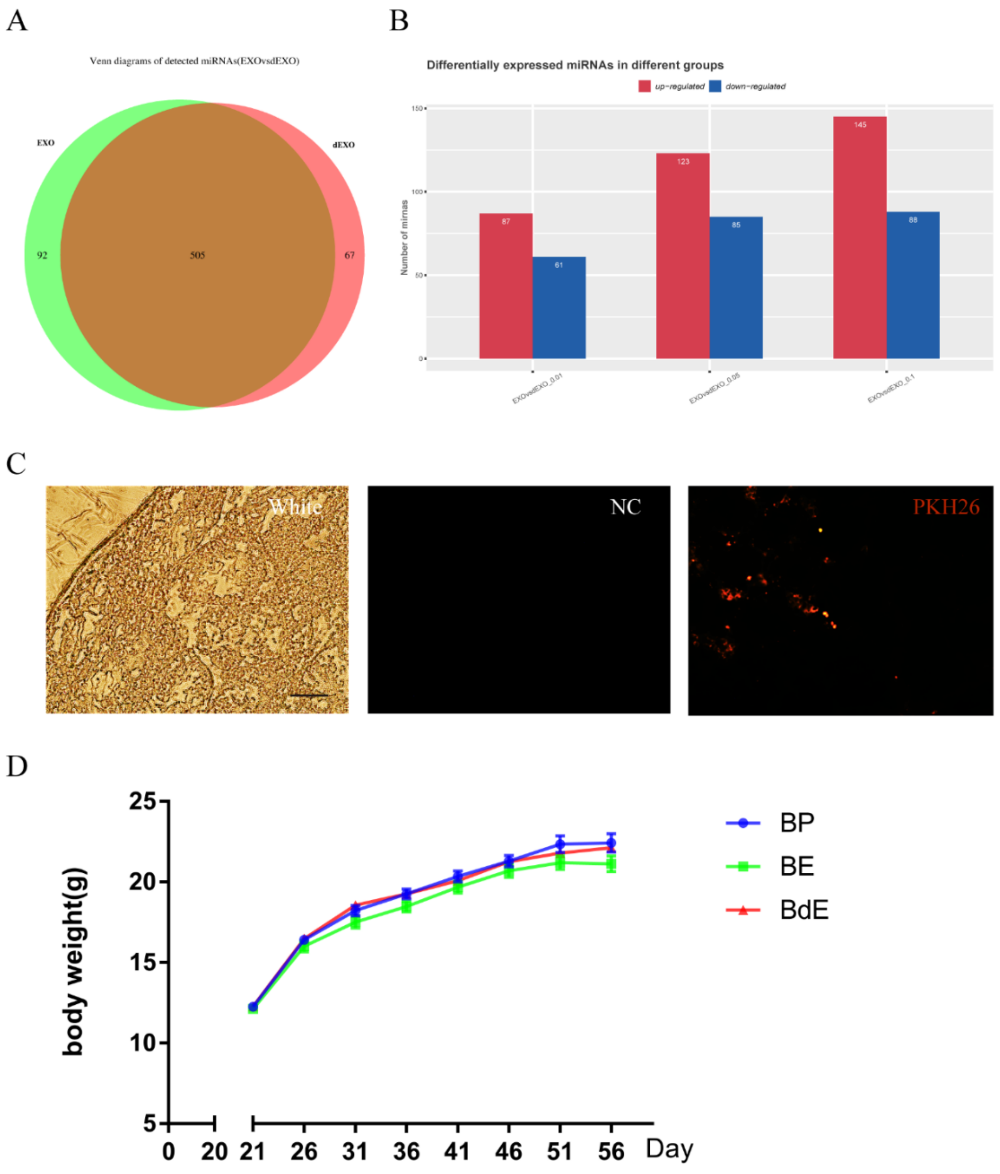

Figure S3. Characteristics of serum exosomes and effects of serum exosomes on busulfan-treated mice. (Data are expressed as means ± SEM.)

(A) Venn diagrams of miRNAs detected in WT_serum exosomes (EXO) and An_serum exosomes (dEXO).

(B) Histogram of differentially expressed miRNAs in EXO and dEXO.

(C) Determination of PKH26 labelled exosomes by fluorescence signals in testicular tissue sections. Scale bar = 100 μm.

(D) Statistical analysis of body weights (n = 8).

Cell type	Marker Genes
Innate Lymph cell	Id2, Il7r, Rora, Thy1, Ccl5, Cd52
Macrophage cell	Apoe, Dab2, Cd74, Adgre1(F4/80)
Endothelial cell	Vwf, Tie1, Tek, Ly6c1
Leydig cell	Cyp17a1, Cyp11a1, Star, Hsd3b, INSL3, Vcam1, Cyp17a1, Hsd3b6, IGF1, SF1
Sertoli cell	Amhr2, Sox9, Rhox, Wt1, Rhox5, Sox8, Gata1, Tfcp2, Cst9
Spermatogonia	DAZL, Uchl1, Crabp1, Stra8, STK31, ID4, Esx1, Ccnd2 , Cenpa , Sohlh1, Sohlh2, Nanos3, Figla, Utf1, Sox3
Spermatocytes	Gpat2, Meioc, Piwil2, Tdrd1, Hormad, sycp1, sycp3, Myl7, Piwil1, Pttg1, Spag6, Tbpl1, Clgn, Pou5f2, Cdk1, Syce1, Syce3, Meiob, Gm960, Psma8, Jag1, Ccne1
Spermatids	Prm1, Prm2, Tnp1, Tnp2, Fam71b, p57kip2, Izumo3, Prss42, Tssk1, Acrv1, Spaca1, Tsga8, Tex36, Sun5, Cstl1, Spag6

Figure S4. Marker genes of each cell subtype.

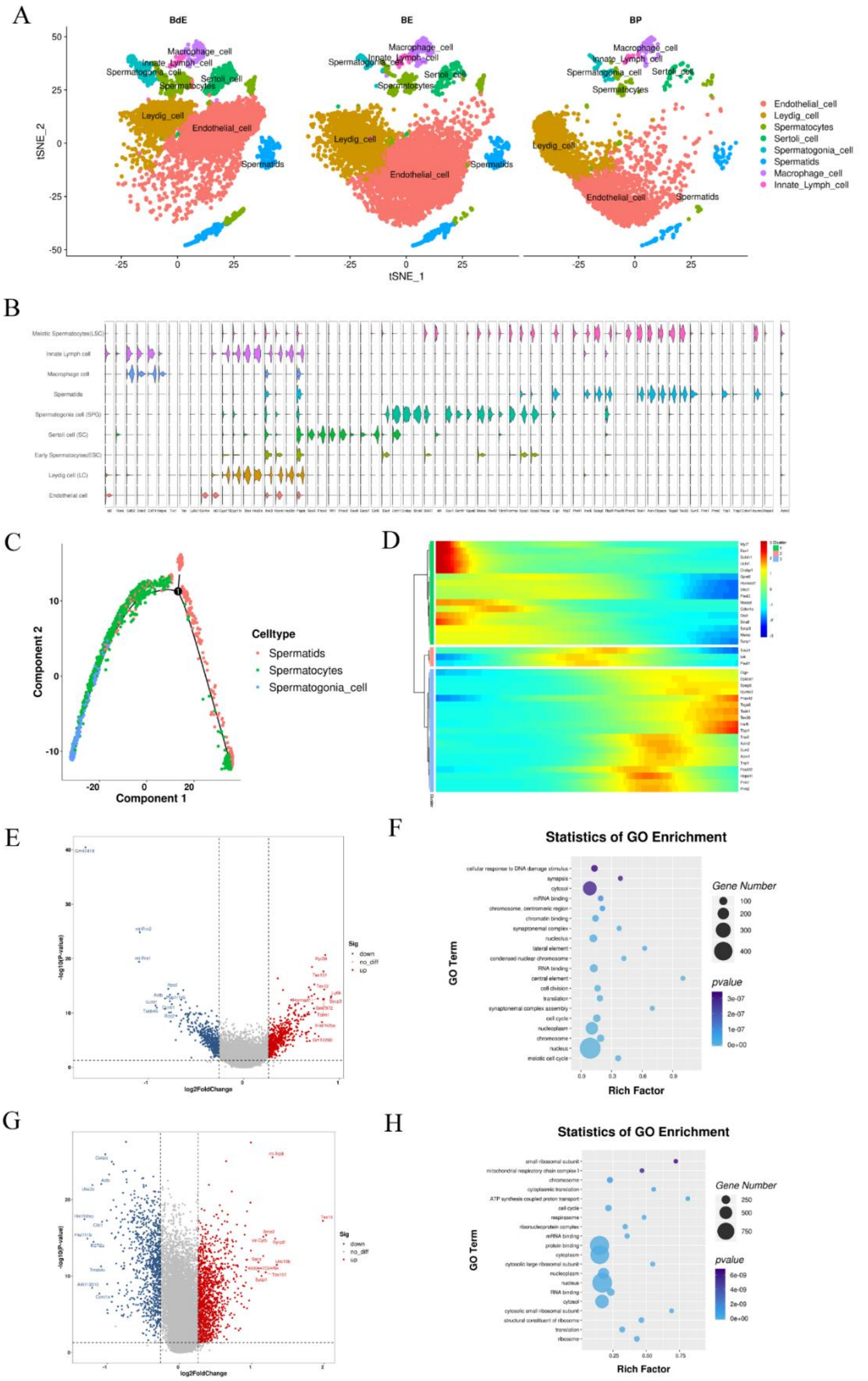

Figure S5. Single-cell transcriptome sequencing of testicular tissue from different treatment groups.

(A) tSNE plot of germ cell clusters from the testes of different treatment groups defined using scRNA-seq analysis.

(B) Vlnplot representation of the average expression of marker genes for each cell population.

(C) Single-cell trajectories of germ cell subsets are shown, with cells ordered in pseudo-time.

(D) DEGs among different cell types are shown as a heatmap.

(E–F) Volcanic map analysis (a) and GO analysis (b) of the DEGs in the SPG cluster between the BE and BP groups. The top 20 GO biological pathways are shown based on the FDR p-values.

(G–H) Volcanic map analysis (a) and GO analysis (b) of the DEGs in the SPC cluster between the BE and BP groups. The top 20 GO biological pathways are shown based on the FDR p-values.

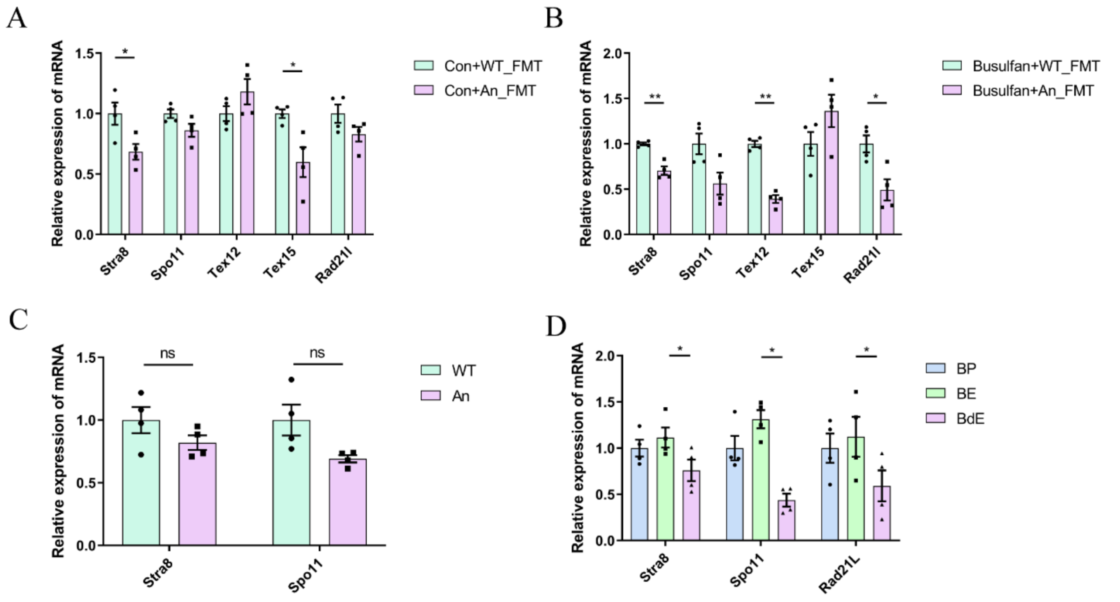

Figure S6. Effect of gut microbiota dysbiosis on meiosis in the mouse testes. (Data are expressed as means ± SEM.)

(A–B) Quantification of meiosis-related mRNA expression levels using qPCR in the testes from the Con+FMT (A) and Busulfan+FMT (B) groups (n = 4).

(C) Quantification of meiosis-related mRNA expression levels by qPCR in the testes of the WT and An group (n = 4).

(D) Quantification of meiosis-related mRNA expression levels using qPCR in the testes from the BP, BE and BdE groups (n = 3).

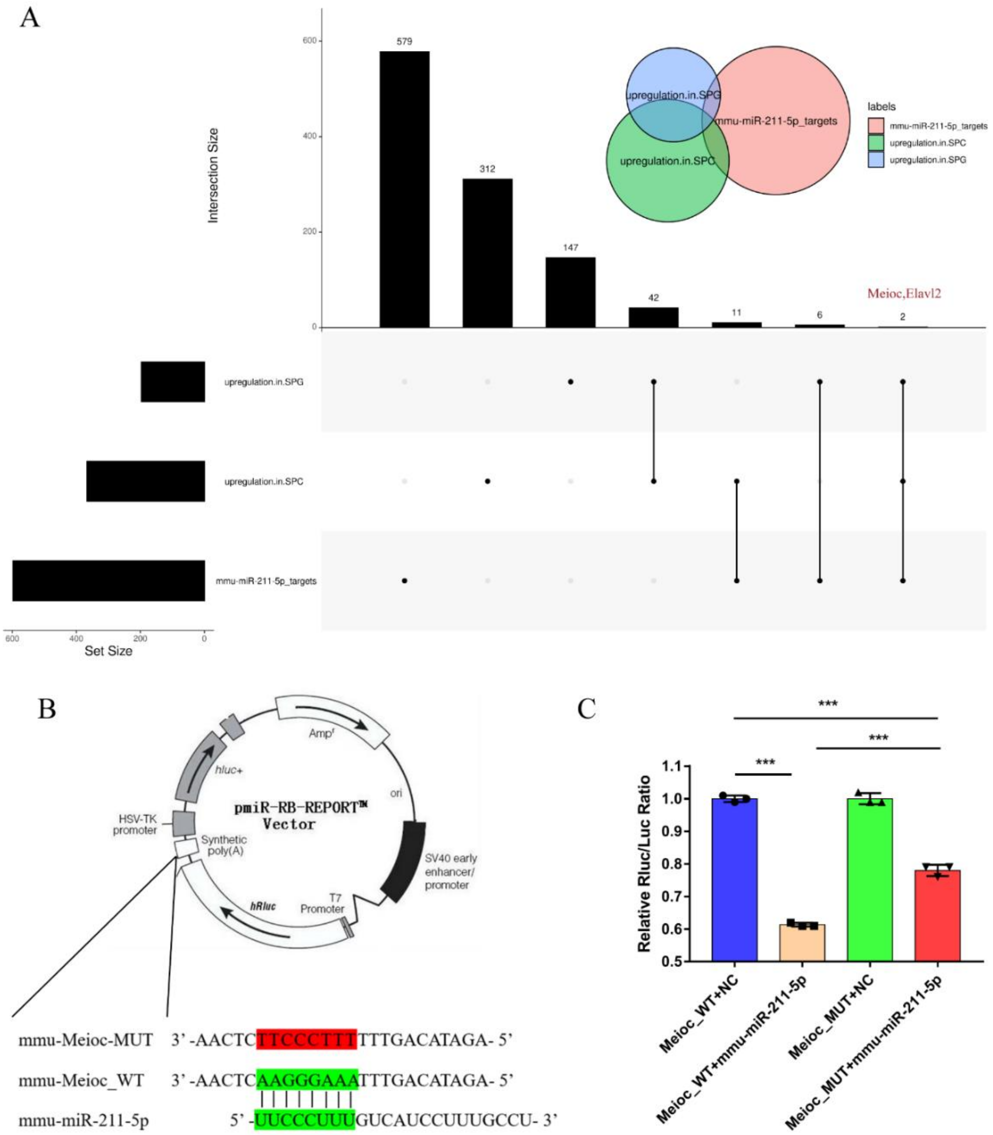

Figure S7. Clarification of the interaction relationship between miR-211-5p and Meioc. (Data are expressed as means ± SEM.)

(A) Intersection of miR-211-5p predicted target genes and DEGs that were simultaneously upregulated in both SPG and SPC cell clusters between the BE and BdE groups.

(B–C) Detection of the interaction relationship using a double-luciferase reporter system. (B) Experimental scheme for the reporter construction. (C) Dual luciferase reporter activities after transfecting 239T cells with miR-211-5p and reporter carrying the 3′-UTR in the long form of Meioc (n = 3).

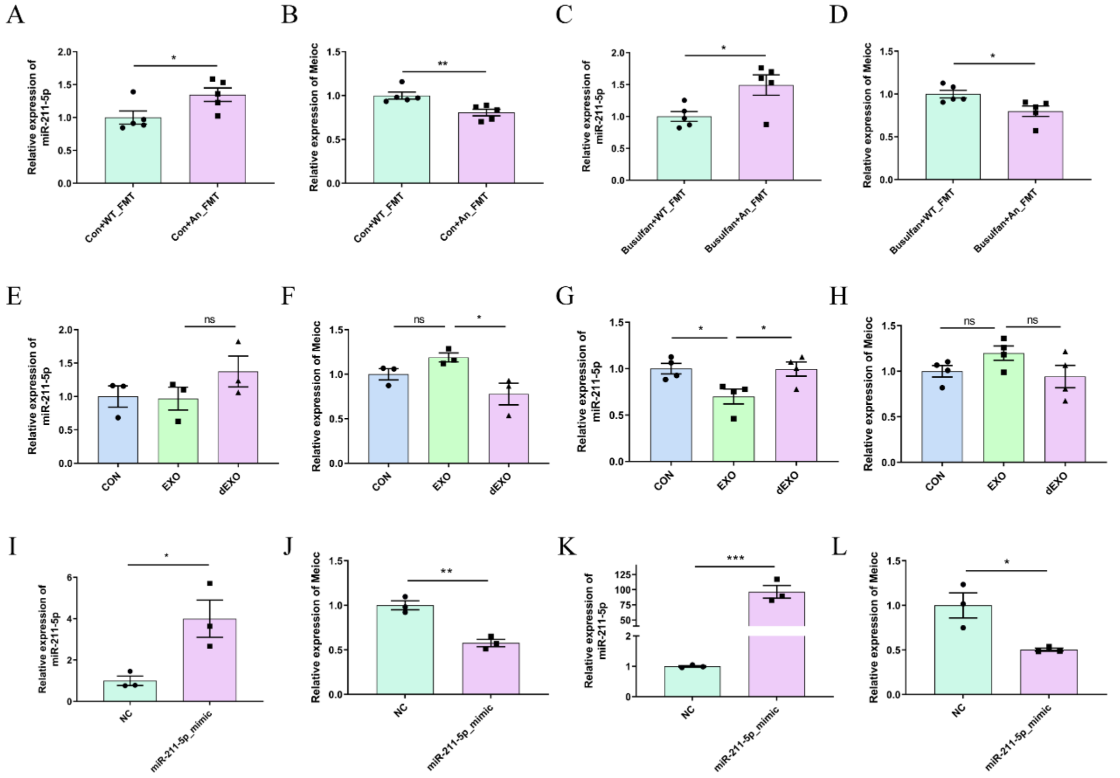

Figure S8. Increased miR-211-5p in circulating exosomes targets Meioc to impair meiosis. (Data are expressed as means ± SEM.)

(A–B) mRNA/miRNA quantification by qPCR in WT+FMT mouse testes (n = 5).

(C–D) mRNA/miRNA quantification by qPCR in Busulfan+FMT mouse testes (n = 5).

(E–F) mRNA/miRNA quantification by qPCR in GC1-SPG cell lines treated with different exosomes (n = 3).

(G–H) mRNA/miRNA quantification by qPCR in the GC2-SPC cell line treated with different exosomes (n = 4).

(I–J) mRNA/miRNA quantification by qPCR in GC1-SPG cell lines treated with 5 nM miRNA mimic (n = 3).

(K–L) mRNA/miRNA quantification by qPCR in the GC2-SPC cell line treated with 5 nM miRNA mimic (n = 3).

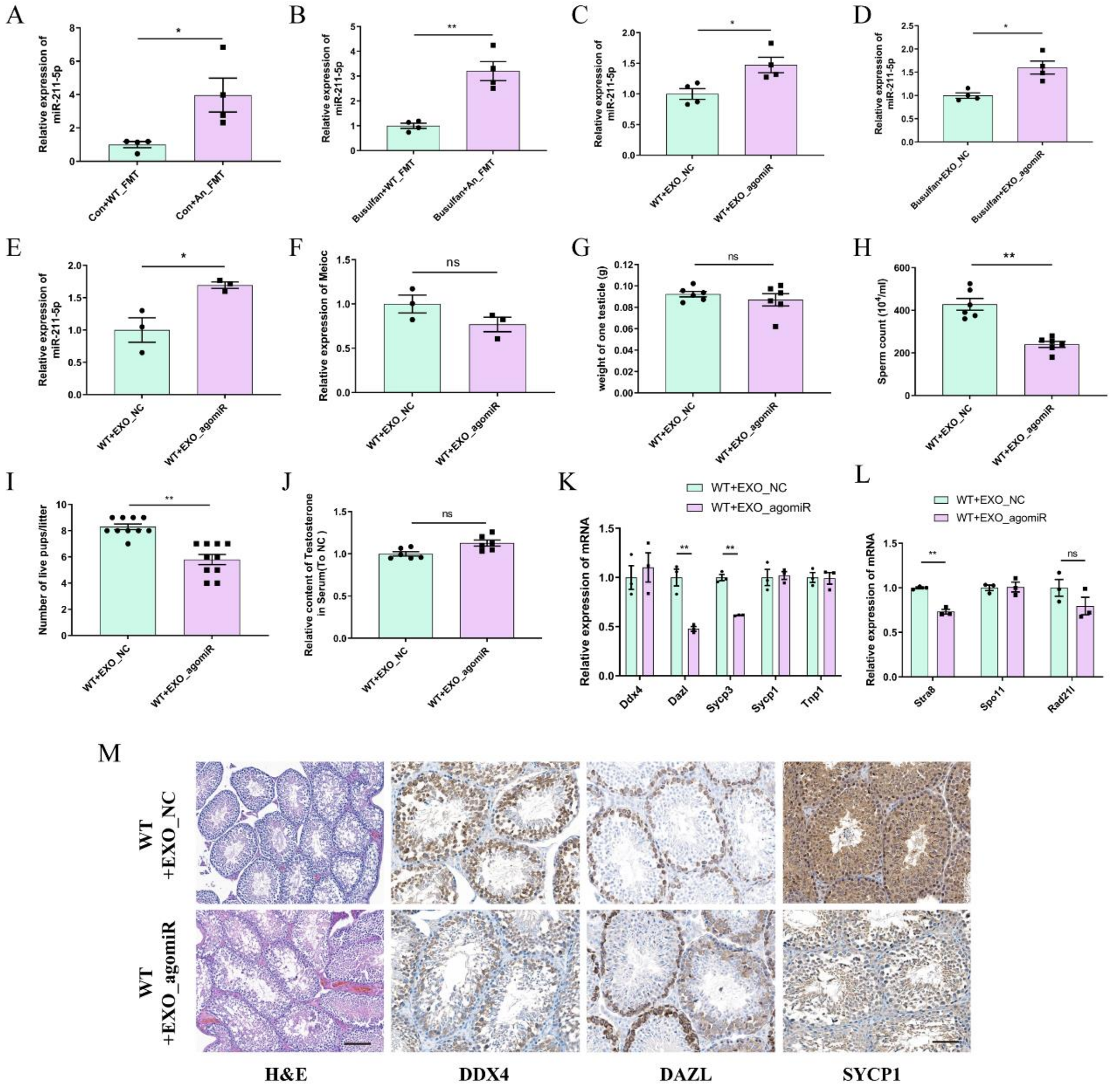

Figure S9. MiR-211-5p loaded in exosomes targets Meioc to impair spermatogenesis (Data are expressed as means ± SEM.)

(A–D) miRNA quantification by qPCR in circulating exosomes from different groups. (A) WT+FMT. (B) Busulfan+FMT. (C) WT+EXO_NC/agomiR. (D) Busulfan+EXO_NC/agomiR (n = 4).

(E–F) mRNA/miRNA quantification by qPCR in WT+EXO_NC/agomiR mouse testes (n = 5).

(G) Statistical analysis of the unilateral testis weights in WT+EXO_NC/agomiR mice (n = 6).

(H) Statistical analysis of sperm counts in WT+EXO_NC/agomiR mice (n = 6).

(I) Statistical analysis of litter sizes in WT+EXO_NC/agomiR group (n = 10).

(J) ELISA analysis of serum testosterone from WT+EXO_NC/agomiR group (n = 6).

(K) Quantification of mRNA by qPCR in the WT+EXO_NC/agomiR mice testes (n = 4).

(L) Quantification of meiosis-related mRNA expression levels using qPCR in the WT+EXO_NC/agomiR group (n = 3).

(M) Representative testicular sections from different treatment groups of WT+EXO_NC/agomiR mice with H&E and immunohistochemistry (IHC) staining. H&E scale bar = 200 μm; IHC scale bar = 100 μm.

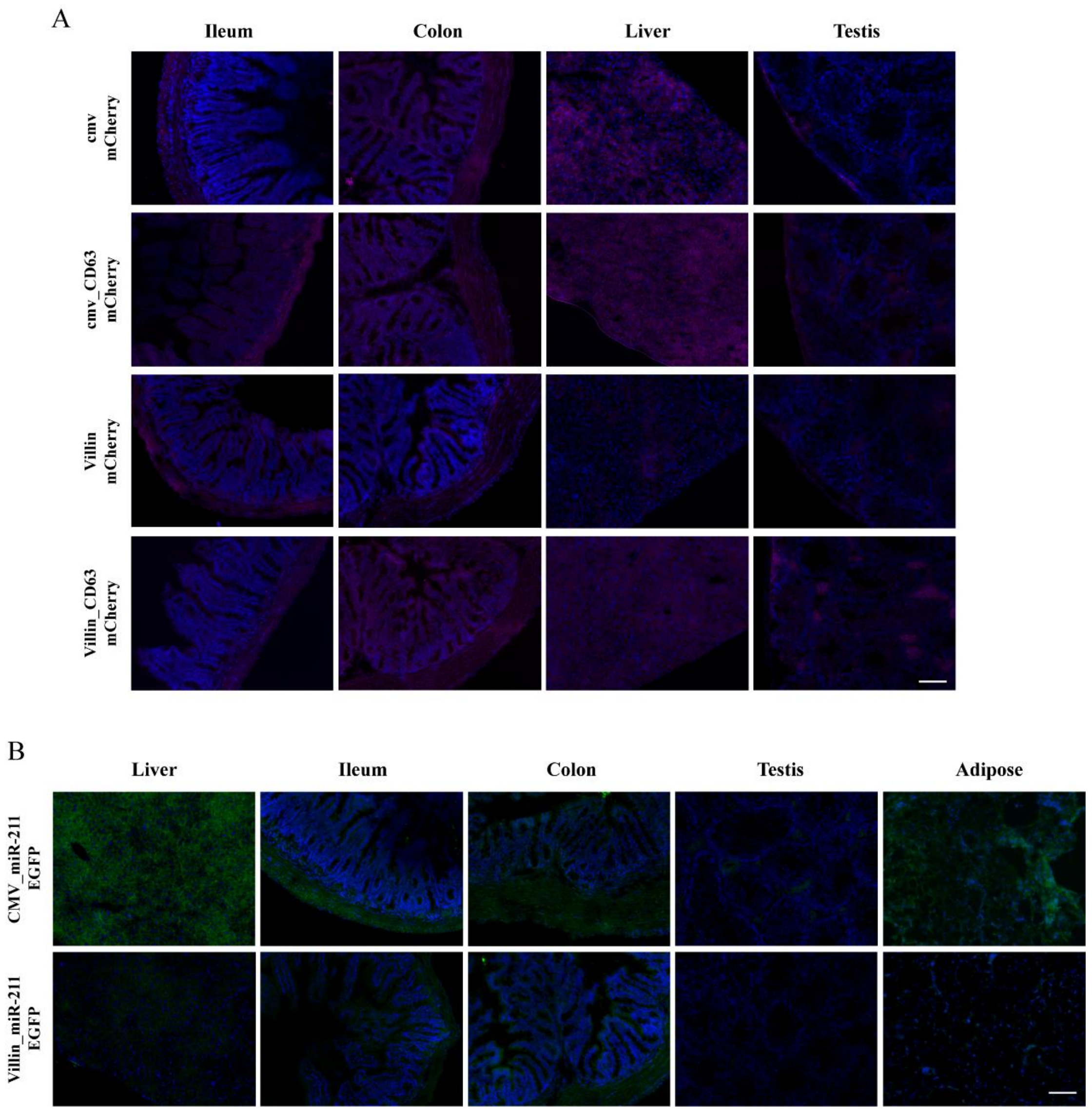

Figure S10. Gut-derived exosomes were transported to testicular tissue.

(A) Representative frozen sections of different tissues expressing mCherry-labeled CD63. Scale bar = 50 μm. (CMV_mCherry, CMV promoter and mCherry fluorescent, fluorescence was detected in tissues of the intestine, liver and testicular mesenchyme; CMV_CD63 mCherry, CMV promoter and mCherry fluorescent labeled CD63 protein, fluorescence was detected in tissues of the intestine, liver and testes; Villin_mCherry, Villin promoter and mCherry fluorescent protein, fluorescence is only detected in intestinal tissue; Villin_CD63 mCherry, Villin promoter and mCherry fluorescent

labeled CD63 protein, fluorescence was detected in tissues of the intestine, testis, and liver.)

(B) Representative frozen sections of different tissues expressing mCherry-labeled CD63. Scale bar = 50 μm. (CMV_miR-211 EGFP, CMV promoter, EGFP fluorescent protein and pre-miR-211, fluorescence was detected in tissues of the intestine, liver and adipose; Villin_miR-211 EGFP, Villin promoter, EGFP fluorescent protein and pre-miR-211, fluorescence is only detected in intestinal tissue.)

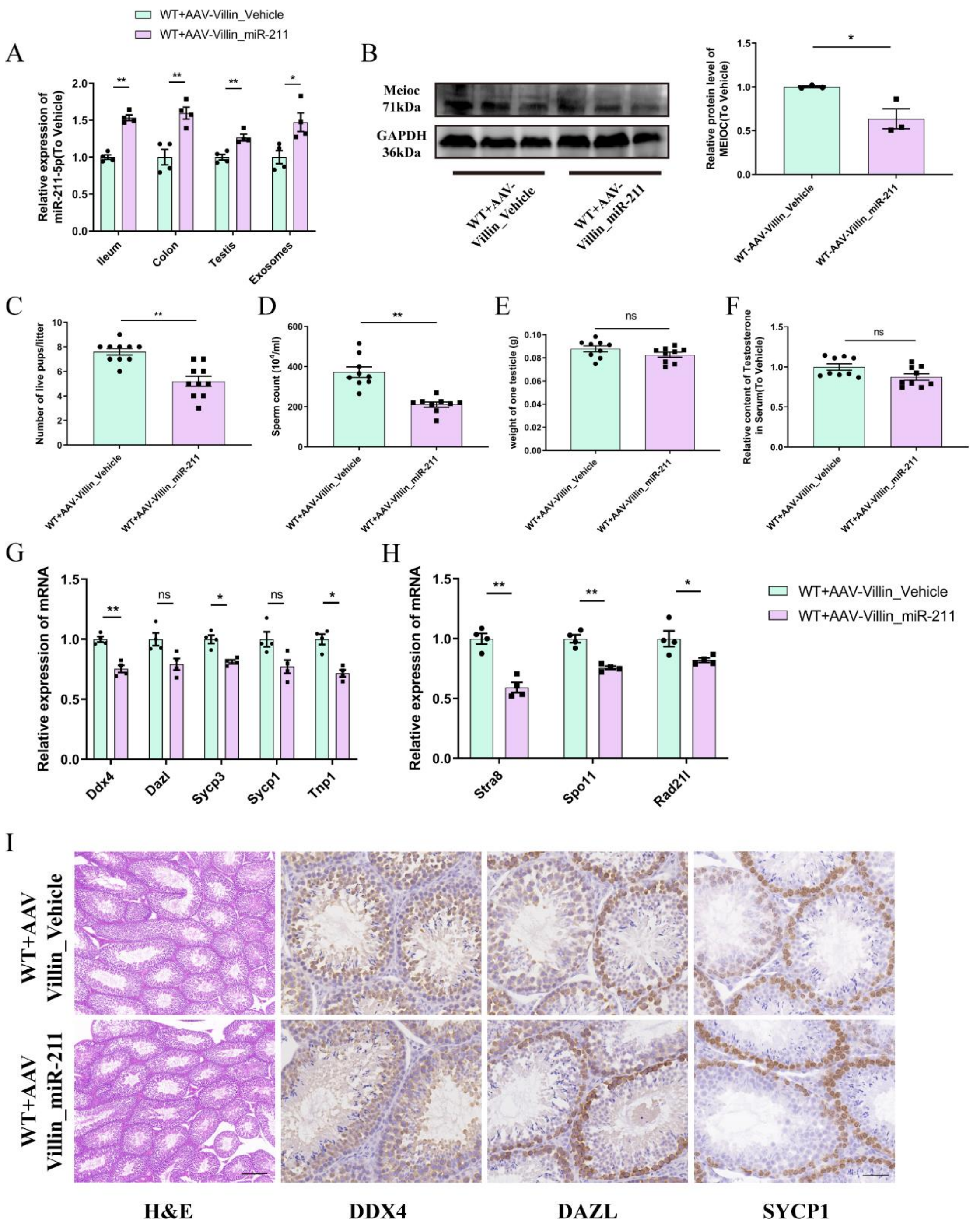

Figure S11. Abnormal elevation of gut-derived miR-211-5p impairs spermatogenesis. (Data are expressed as means ± SEM.)

(A) miRNA quantification using qPCR in the different tissues of WT+AAV Villin_Vehicle/miR-211 (n = 4).

(B) MEIOC protein quantification using western blot in the testes from the WT+AAV Villin_Vehicle/miR-211 groups (n = 3).

(C) Statistical analysis of litter sizes in WT+AAV Villin_Vehicle/miR-211 groups (n = 10).

(D) Statistical analysis of sperm counts in WT+AAV Villin_Vehicle/miR-211 groups (n = 9).

(E) Statistical analysis of unilateral testis weights in WT+AAV Villin_Vehicle/miR-211 groups (n = 9).

(F) ELISA analysis of serum testosterone in WT+AAV Villin_Vehicle/miR-211 groups (n = 9).

(G) Quantification of mRNA by qPCR in the WT+AAV Villin_Vehicle/miR-211 treated mice testes (n = 4).

(L) Quantification of meiosis-related mRNA expression levels by qPCR in WT+AAV Villin_Vehicle/miR-211 groups (n = 4).

Oligonucleotides	Source	5`-3` sequence
Ppia forward	Comate Bioscience Co.,Ltd.	GAAGCCATGGAGCGTTTTGG
Ppia reverse	Comate Bioscience Co.,Ltd.	ATTGCGAGCAGATGGGGTAG
Ddx4 forward	Comate Bioscience Co.,Ltd.	GGTGGTGGCCTTTTTGGTTC
Ddx4 reverse	Comate Bioscience Co.,Ltd.	GGTAAGTGTCACCATTGCCTG
Dazl forward	Comate Bioscience Co.,Ltd.	GGATGGATGAAACCGAAAT
Dazl reverse	Comate Bioscience Co.,Ltd.	ATGCCTGAACATACTGAGTGATA
Sycp3 forward	Comate Bioscience Co.,Ltd.	TAGAATTGTTCAGAGCCAGAG
Sycp3 reverse	Comate Bioscience Co.,Ltd.	TTGCTGCTGAGTTTCCATC
Sycp1 forward	Comate Bioscience Co.,Ltd.	TGAGCAGCAGTCAAGTGTC
Sycp1 reverse	Comate Bioscience Co.,Ltd.	CTGTTCGAGCATGGGCAAA
Tnp1 forward	Comate Bioscience Co.,Ltd.	TTCGGCAGAAAGTACCATG
Tnp1 reverse	Comate Bioscience Co.,Ltd.	TGTTGTTTGAAGACCACCAG
Stra8 forward	Comate Bioscience Co.,Ltd.	GCCTGGAGACCTTTGACGA
Stra8 reverse	Comate Bioscience Co.,Ltd.	GGCTTTTGGAAGCAGCCTTT
Spo11 forward	Comate Bioscience Co.,Ltd.	GCCCAGGAGGAGTCTGCAC
Spo11 reverse	Comate Bioscience Co.,Ltd.	CCAGCAATCAATCCCTTGGA
Tex12 forward	Comate Bioscience Co.,Ltd.	AGTCTCCAGTGCCAGATAGT
Tex12 reverse	Comate Bioscience Co.,Ltd.	AGATTAATTTCCTTGCTCACATCA
Tex15 forward	Comate Bioscience Co.,Ltd.	AGGCAACATTCAAGCATCCA
Tex15 reverse	Comate Bioscience Co.,Ltd.	AGTGAGCCAGGTAGTGATCTTT
Rad21l forward	Comate Bioscience Co.,Ltd.	GGTGAAAATTGCACTCCGAAC
Rad21l reverse	Comate Bioscience Co.,Ltd.	CGAACAACCCCCAAAAGAAG
Meioc forward	Comate Bioscience Co.,Ltd.	GGAAGAACAGGATAAGTCGC
Meioc reverse	Comate Bioscience Co.,Ltd.	TCCACTGCTGACACAGATTC
mmu-miR-211-5p forward	Comate Bioscience Co.,Ltd.	GGGCTTCCCTTTGTCATCCTT
mmu-miR-211-5p reverse	Comate Bioscience Co.,Ltd.	CCAGTGCAGGGTCCGAGGT
mmu-miR-211-5p stem ring	Comate Bioscience Co.,Ltd.	GTCGTATCCAGTGCAGGGTCCGAGG TATTCGCACTGGATACGACAGGCAA

Figure S12. Primers for qPCR.
